# Supplementary material for: Crop damage by vertebrates in Latin America: current knowledge and potential future management directions
Source: PeerJ. 2022 Mar 25;10:e13185. doi: 10.7717/peerj.13185 (PMC8958972; doi:10.7717/peerj.13185)
Supplement: Supplemental Information 1 — Including information on the type of study that they are, their country, whether coordinates and a map of the study area are provided, the coordinates of the locations plotted in Fig. 3, and notes about how these coordinates were obtained. [file peerj-10-13185-s001.docx]

**Crop damage by vertebrates in Latin America: current knowledge and potential future management directions**

**Adrián Alejandro Cuesta Hermira, Fernanda Michalski**

Supplementary Material

**Table S1: List of the 113 reviewed studies with geographical data.**

Including information on the type of study that they are, their country, whether coordinates and a map of the study area are provided, the coordinates of the locations plotted in Figure 3, and notes about how these coordinates were obtained.

| **Study** | **Type of study** | **Country** | **Exact Coordinates** | **Map provided** | **Plotted locations**  **(Latitude, longitude)** | **Notes** |
| --- | --- | --- | --- | --- | --- | --- |
| Abba et al. (2015) | Crop-feeding species behavior | Argentina | No | Yes | -34.4169, -60.4072  -35.4281, -62.1458  -36.6042, -62.9100  -36.4214, -58.5411  -38.3842, -60.2508 | Approximate middle point for each of the regions found using Google Earth, based on provided map. |
| Abrahams et al. (2018) | Crop damage evaluation, Farmer perception | Brazil | No | Yes | -5.5544, -67.7015 | Approximate middle point found using Google Earth, based on provided map. |
| Aguiar et al. (2011) | Crop-feeding species behavior | Brazil | Yes | No | -24.2117, -50.5581 | - |
| Albarracín and Aliaga-Rossel (2018) | Crop damage evaluation, Farmer perception | Bolivia | No | Yes | -16.5669, -67.6094 | Approximate middle point found using GE. |
| de Almeida-Jácomo et al. (2013) | Crop-feeding species behavior | Brazil | Yes | Yes | -18.3167, -52.7500 | - |
| Aris et al. (2008) | Farmer perception | Peru | Yes | No | -13.0614, -73.7769 | Locations within 50 km of each other, Approximate middle point found using Google Earth. |
| Arroyo-Quiroz et al. (2017) | Farmer perception | Mexico | No | Yes | 21.3050, -99.4564 | Approximate location found using GE, based on named reserve. |
| Avery et al. (2001) | Crop protection experiment | Venezuela | No | No | 9.5440, -69.1864 | Ex-situ experiment, approximate location of where birds where captured found using Google Earth. |
| Barceló et al. (2012) | Farmer perception | Mexico | No | Yes | 28.6562, -106.1021  26.8883, -103.9347  24.9104, -104.9132  22.7636, -102.5886 | Approximate locations found using GE, based on named places. |
| Basili and Temple (1999a) | Crop damage evaluation, Farmer perception | Venezuela | No | No | 9.0950, -69.0992 | Approximate location found using Google Earth, based on named region. |
| Basili and Temple (1999b) | Crop-feeding species behavior, Farmer perception | Venezuela | No | Yes | 9.0950, -69.0992 | Approximate location found using Google Earth, based on named region. |
| Berón et al. (2020) | Crop damage evaluation | Argentina | Yes | Yes | -31.5550, -60.6769 | - |
| Bou et al. (2016) | Crop damage evaluation | Uruguay | No | Yes | -31.9892, -58.1075  -32.5636, -57.9856  -33.2633, -58.0219  -34.2083, -57.6803  -34.5911, -56.6089 | Approximate locations found using Google Earth, based on provided map. |
| Boulton et al. (1996) | Crop damage evaluation, Farmer perception | Barbados | No | Yes | 13.1882, -59.5353 | Country-wide study, used central point for Barbados. |
| Bourne (1981) | Crop-feeding species behavior | Guyana | Yes | No | 6.4667, -57.7500 | - |
| Bruggers et al. (1998) | Pest species or outbreak overview | Argentina, Uruguay | No | Yes | -31.2167, -57.9333 | Approximate location found using GE, based on named region. |
| Bucher and Aramburú (2014) | Crop-feeding species behavior | Argentina | No | Yes | -36.2678, -60.3192 | Approximate location found using GE, based on named region. |
| Bucher and Ranvaud (2006) | Pest species or outbreak overview | Argentina, Colombia, Uruguay, Bolivia, Brazil | No | No | -32.2953, -63.5825  -32.5169, -59.1042  -33.8761, -66.2375  -26.5847, -60.9542  -25.2489, -64.7183  3.8025, -76.6431  -17.8131, -63.1575  -23.5528, -46.6411  -25.2406, -52.0297 | Approximate locations found using GE, based on named regions. |
| Calamari et al. (2018) | Crop-feeding species behavior | Argentina | No | Yes | -31.6181, -60.7063 | Approximate middle point of study region found using GE. |
| Canavelli et al. (2012) | Pest species or outbreak overview,  Protection technique evaluation | Argentina | No | Yes | -38.4530, -63.5989 | Country-wide study, used central point for Argentina. |
| Canavelli et al. (2013) | Farmer perception | Argentina | No | No | -31.6125, -60.0783 | Approximate location found using Google Earth, based on named region. |
| Canavelli et al. (2014) | Crop damage evaluation | Argentina | No | Yes | -31.6125, -60.0783 | Approximate location found using GE, based on named region. |
| Can-Hernandez et al. (2019) | Farmer perception, Crop damage evaluation | Mexico | No | Yes | 17.5911, -92.4550 | Locations within 50 km of each other, approximate middle point found using Google Earth. |
| de Carvalho et al. (2019) | Farmer perception | Brazil | No | Yes | -21.1311, -44.2533 | Approximate middle point found using Google Earth, based on provided map. |
| Castilho et al. (2018) | Farmer perception | Brazil | No | Yes | -15.1636, -39.3458 | Approximate location found using GE, based on named protected area. |
| Castillo-Chinchilla et al. (2018) | Farmer perception | Costa Rica | Yes | Yes | 10.1900, -85.3644 | - |
| Castillo-Lopez et al. (2017) | Crop protection experiment, Farmer perception,  Crop damage evaluation | Colombia | No | No | 5.0773, -73.4215 | Locations within 50 km of each other, approximate middle point found using Google Earth, based on named cities. |
| Cervo and Guadagnin (2020) | Crop-feeding species behavior | Brazil | No | Yes | -16.7197, -56.8389  -28.6318, -51.5735  -28.7965, -51.0955  -29.0479, -50.1435  -29.4419, -50.5797  -29.7914, -55.7813  -30.8722, -55.5208  -30.9734, -54.6670  -30.5459, -52.5247 | Approximate locations found using Google Earth, based on named cities. |
| Chaves and Bicca-Marques (2017) | Crop damage evaluation, Crop-feeding species behavior, Farmer perception | Brazil | Yes | Yes | -30.1985, -51.0915 | Locations within 50 km of each other, approximate middle point found using Google Earth. |
| Cirne and López-Iborra (2005) | Crop-feeding species behavior | Brazil | Yes | No | -32.2000, -52.5833 | - |
| Codesido et al. (2015) | Crop-feeding species behavior | Argentina | No | Yes | -36.2678, -60.3192 | Approximate location found using GE, based on named region. |
| Cornejo (2000) | Crop damage evaluation, Crop protection experiment | Mexico | No | No | 19.54205, -96.884908 | Approximate location found using GE, based on named city. |
| Corrêa et al. (2018) | Crop-feeding species behavior | Brazil | Yes | Yes | -30.2392, -51.0897 | - |
| Cossios et al. (2018) | Farmer perception | Peru | Yes | Yes | -10.4076, -76.3943 | Locations within 50 km of each other, approximate middle point found using Google Earth. |
| Costán & Sarasola (2017) | Crop-feeding species behavior | Argentina | Yes | No | -36.9136, -64.2614 | - |
| Dardanelli et al. (2016) | Crop-feeding species behavior | Argentina | No | Yes | -32.5169, -59.1042 | Approximate location found using GE, based on named region. |
| Dore et al. (2018) | Farmer perception | Saint Kitts and Nevis | No | Yes | 17.3154, -62.7428 | Island-wide study, approximate middle-point found using GE. |
| Doutel-Ribas et al. (2019) | Protection technique evaluation, Crop-feeding species behavior | Brazil | Yes | Yes | -21.5454, -54.2273 | Locations within 50 km of each other, Approximate middle point found using Google Earth. |
| Eiris and Barreto (2009) | Crop-feeding species behavior | Venezuela | Yes | No | 8.8453, -67.5428 | - |
| Engeman et al. (2010) | Farmer perception, Crop damage evaluation | Puerto Rico | No | No | 18.0383, -67.0061 | Approximate location found using GE, based on named region. |
| Escobar-Lasso et al. (2020) | Farmer perception, Crop damage evaluation | Colombia | No | Yes | 4.7283, -75.6361 | Approximate locations found using Google Earth, based on provided map. |
| Felix et al. (2014) | Crop damage evaluation, Crop-feeding species behavior,  Farmer perception | Brazil | Yes | Yes | -22.2217, -54.8064 | - |
| Ferraz et al. (2003) | Crop damage evaluation | Brazil | Yes | Yes | -22.7083, -47.6417 | - |
| Ferraz et al. (2007) | Crop-feeding species behavior | Brazil | No | Yes | -22.7083, -46.9667 | Approximate middle point of study region found using GE, based on provided map. |
| Ferraz et al. (2009) | Crop-feeding species behavior | Brazil | No | Yes | -22.7083, -46.9667 | Approximate middle point of study region found using GE, based on provided map. |
| Figueroa (2013) | Crop-feeding species behavior | Peru, Venezuela, Ecuador, Colombia, Bolivia | Yes | Yes | -5.5358, -79.7628  -6.3678, -78.0022  -6.1392, -78.7250  -6.3531, -79.4814  -6.6978, -79.3594  -9.1528, -77.6475  -10.3258, -75.3889  -10.6569, -75.0178  -12.2617, -71.2844  -13.1831, -71.6181  -13.2261, -72.4950  -13.3886, -72.8817  -12.7278, -70.9950  -13.5219, -69.5706 | - |
| Flores-Armillas et al. (2020) | Farmer perception, Crop damage evaluation | Mexico | No | Yes | 18.4640, -98.9731 | Approximate middle point found using Google Earth. |
| de Freitas et al. (2008) | Crop-feeding species behavior | Brazil | No | No | -20.5125, -47.3083 | Approximate middle point found using Google Earth. |
| Fuentes and Campusano (1985) | Pest species or outbreak overview, Farmer perception | Chile | No | No | -30.0322, -70.7081  -29.9022, -71.2519 | Approximate locations found using GE, based on named cities. |
| Galetti (1993) | Crop-feeding species behavior | Brazil | Yes | No | -22.7833, -49.1167 | - |
| García and Peiró (2016) | Crop-feeding species behavior, Crop damage evaluation | Cuba | Yes | Yes | 22.3634, -80.557167 | - |
| García-Mendoza and Prieto-Rosales (2019) | Crop damage evaluation | Peru | Yes | No | -12.4083, -74.6767 | - |
| Gonzalez and Acosta-Perez (2002) | Crop damage evaluation | Mexico | No | No | 18.8729, -98.9141 | Approximate location found using GE, based on named city. |
| Gorosábel et al. (2019) | Crop damage evaluation | Argentina | Yes | No | -38.3739, -60.2797  -38.3480, -59.6183 | - |
| Hilje (1992) | Pest species or outbreak overview | Costa Rica | No | No | 9.9168, -84.0743 | Country-wide study, used central point for Costa Rica |
| Horrocks and Baulu (1988) | Protection technique evaluation | Barbados | No | No | 13.1882, -59.5353 | Country-wide study, used central point for Barbados. |
| Horrocks and Baulu (1994) | Farmer perception, Crop damage evaluation | Barbados | No | No | 13.1882, -59.5353 | Country-wide study, used central point for Barbados. |
| Horváth et al. (2001) | Crop-feeding species behavior | Mexico | No | Yes | 16.1086, -91.6992 | Approximate location found using GE, based on named protected area. |
| Ibañez et al. (2016) | Crop damage evaluation | Argentina | Yes | No | -34.8833, -58.0667 | - |
| Jackson (1988) | Pest species or outbreak overview | Argentina | No | No | -38.4530, -63.5989 | Country-wide study, used central point for Argentina. |
| Key and de la Piedra Constantino (1992) | Protection technique evaluation | Mexico | No | Yes | 16.7460, -93.1296  16.2351, -93.2563  16.2327, -92.1304  14.9114, -92.2780 | Approximate locations found using GE, based on named cities. |
| Lima et al. (2019) | Pest species or outbreak overview, Farmer perception | Brazil | No | Yes | -12.6764, -56.9236 | State-wide study, used approximate central point of Mato Grosso, found using Google Earth. |
| Lins and Ferreira (2018) | Crop-feeding species behavior | Brazil | Yes | No | -7.5164, -34.9203 | Error in provided coordinates, approximate location found using Google Earth, based on mentioned city. |
| Lobão and Nogueira-Filho (2011) | Crop damage evaluation, Farmer perception | Brazil | Yes | No | -15.0308, -39.1611 | Locations within 50km of each other, approximate middle point found using Google Earth. |
| López-Torres et al. (2012) | Crop-feeding species behavior | Puerto Rico | Yes | Yes | 18.3915, -65.8611 | Locations within 50 km of each other, approximate middle point found using GE. |
| Loza-del-Carpio et al. (2016) | Crop damage evaluation | Peru | Yes | No | -15.2333, -70.7167 | - |
| MacGregor-Fors et al. (2011) | Pest species or outbreak overview | Mexico | No | Yes | 26.0117, -111.3478  19.7061, -101.1950  20.5278, -100.8111  19.4328, -99.1331  19.0414, -98.2064  17.0731, -96.7264  16.7517, -93.1031 | Approximate locations found using GE, based on named cities. |
| Marchand (2016) | Farmer perception | Brazil | No | Yes | -2.4546, -58.2689 | Locations within 50 km of each other, Approximate middle point found using Google Earth, based on provided map. |
| McKinney (2011) | Crop-feeding species behavior | Costa Rica | Yes | Yes | 9.7956, -84.9208 | - |
| McKinney (2019) | Crop-feeding species behavior | Costa Rica | No | Yes | 9.7956, -84.9208 | Coordinates taken from another study by the same author in the same wildlife refuge. |
| Melo and Cheschini (2012) | Crop damage evaluation | Brazil | No | No | -18.9488, -48.2174 | Approximate location found using GE, based on named place |
| Mendonça et al. (2011) | Farmer perception | Brazil | Yes | Yes | -7.0767, -36.0611 | - |
| Mitchell and Bruggers (1985) | Crop damage evaluation, Crop protection experiment | Dominican Republic | No | No | 19.2981, -70.2564 | Approximate location found using Google Earth, based on named city. |
| Monge (1999) | Crop damage evaluation | Costa Rica | No | Yes | 9.9168, -84.0743 | Country-wide study, used central point for Costa Rica. |
| Monge (2013) | Pest species or outbreak overview | Costa Rica | No | No | 9.9168, -84.0743 | Country-wide study, used central point for Costa Rica. |
| Monge-Meza (2011) | Pest species or outbreak overview | Costa Rica | No | Yes | 10.2489, -83.6392 | Approximate location found using Google Earth, based on named region. |
| Monge-Meza and Orozco (2010) | Crop damage evaluation | Costa Rica | Yes | No | 11.0667, -85.5833 | - |
| Monge-Meza et al. (2014) | Crop damage evaluation | Costa Rica | Yes | No | 10.1833, -84.2667 | - |
| Naughton-Treves et al. (2003) | Crop-feeding species behavior, Crop damage evaluation | Peru | No | Yes | -12.6564, -69.2710 | Approximate location found using Google Earth, based on provided map. |
| Olivera et al. (2016) | Protection technique evaluation | Uruguay | No | No | -34.9008, -56.1644 | Approximate locations found using GE, based on named facilities. |
| Parra et al. (2012) | Crop damage evaluation | Venezuela | Yes | No | -8.7500, -67.5333 | - |
| Pedrana et al. (2014) | Crop-feeding species behavior | Argentina | No | Yes | -38.5950, -61.4628 | Approximate locations found using GE, based on provided map. |
| Pedrosa et al. (2015) | Pest species or outbreak overview | Brazil | No | Yes | -19.1533, -49.1394 | Country-wide study, used central point for Southern Brazil |
| Pereira et al. (2019) | Farmer perception | Brazil | No | Yes | -22.2971, -44.7009 | Locations within 50km of each other, approximate middle point found using Google Earth. |
| Pérez and Bulla (2000) | Crop-feeding species behavior | Venezuela | Yes | No | 8.9981, -65.7425 | - |
| Pérez and Pacheco (2006) | Crop protection experiment, Crop damage evaluation | Bolivia | No | Yes | -16.2034, -67.8367 | Approximate middle point found using Google Earth, based on named town. |
| Pérez and Pacheco (2014) | Crop protection experiment, Crop damage evaluation | Bolivia | No | No | -16.1986, -67.8994 | Approximate middle point found using Google Earth, based on places named in article. |
| Peyton (1980) | Crop-feeding species behavior | Peru | No | Yes | -7.9428, -77.6675  -10.8197, -75.5992  -13.6642, -70.4839 | Approximate locations found using GE, based on provided map |
| Poleo et al. (2010) | Crop-feeding species behavior | Venezuela | No | No | 8.6472, -67.1897 | Approximate location found using GE, based on named region. |
| Ranvaud et al. (2001) | Crop-feeding species behavior | Brazil | Yes | Yes | -22.7833, -50.5833 | - |
| Renfrew and Saavedra (2007) | Crop-feeding species behavior, Farmer perception | Bolivia | Yes | Yes | -17.2186, -62.8952  -17.1133, -63.9375  -14.8797, -64.8525 | Locations in each area within 50km of each other, approximate middle points found using Google Earth, based on study site coordinates. |
| Renfrew et al. (2017) | Crop-feeding species behavior | Argentina, Bolivia | Yes | No | -15.7610, -64.1570  -25.9240, -58.5350 | - |
| Robles et al. (2003) | Crop damage evaluation, Crop protection experiment | Peru | Yes | No | -12.1167, -75.2000 | - |
| Rocha and Fortes (2015) | Farmer perception | Brazil | Yes | No | -29.4472, -53.2806 | - |
| Rodriguez and Avery (1996) | Pest species or outbreak overview | Uruguay | No | No | -32.5228, -55.7672 | Country-wide study, used central point for Uruguay. |
| Rodriguez et al. (1995) | Crop protection experiment, Crop damage evaluation | Uruguay | No | No | -34.1209, -57.7018  -32.6984, -57.6357 | Approximate locations found using GE, based on named cities. |
| Rodriguez et al. (2004) | Farmer perception, Crop damage evaluation | Uruguay | No | No | -34.5425, -55.9434 | Approximate location found using GE, based on named region. |
| Romero-Balderas et al. (2006) | Crop damage evaluation, Farmer perception | Mexico | No | Yes | 16.1370, -90.8916 | Locations within 50 km of each other, Approximate middle point found using Google Earth, based on provided map. |
| Rosa et al. (2018) | Protection technique evaluation | Brazil | Yes | Yes | -22.3500, -44.7833  -30.8833, -55.5167 | - |
| Sanchez et al. (2016) | Crop damage evaluation | Argentina | No | Yes | -41.0461, -62.8730 | Approximate location found using Google Earth, based on provided map. |
| Sanchez-Cordero and Martinez-Meyer (2000) | Pest species or outbreak overview | Mexico | No | Yes | 18.4572, -95.3997 | Study involves the whole state, location of central point of the state. |
| Santos (2018) | Pest species or outbreak overview, Farmer perception | Brazil | No | No | -10.3326, -36.8667 | Approximate location found using Google Earth, based on named region. |
| Saucedo et al. (2010) | Farmer perception, Crop damage evaluation | Cuba | No | Yes | 22.4950, -79.9206 | Approximate location found using GE, based on named region. |
| Silva-Andrade et al. (2016) | Farmer perception | Brazil | Yes | Yes | -8.7117, -36.4150 | - |
| Silva-Rodríguez et al. (2006) | Farmer perception | Chile | Yes | No | -40.2333, -73.0667 | - |
| Spagnoletti et al. (2017) | Farmer perception, Crop damage evaluation | Brazil | Yes | Yes | -9.6313, -45.4303 | Locations within 50km of each other, approximate middle point found using Google Earth. |
| Trivedi et al. (2004) | Crop damage evaluation | Peru | Yes | No | -12.6508, -68.9278 | - |
| Valencia (1980) | Crop damage evaluation, Crop protection experiment | Colombia | No | Yes | 12.5405, -81.7043  2.9683, -78.1844  1.7874, -78.7648  11.2724, -73.3093 | Approximate locations found using GE, based on provided map. |
| Valencia et al. (1994) | Pest species or outbreak overview | Colombia | No | Yes | 12.5405, -81.7043  1.7569, -78.4639  2.3992, -71.4950  5.0567, -72.8864  8.5331, -76.0842  8.9544, -73.9036 | Approximate locations found using GE, based on provided map. |
| Villa et al. (1998) | Crop-feeding species behavior | Mexico | No | No | 18.2386, -96.1417 | Approximate middle point found using GE. |
| Villafana-Martin et al. (1999) | Crop protection experiment | Costa Rica | No | No | 9.91681, -84.07426 | No location information provided. Used central point for Costa Rica, found using Google Earth. |
| del Villar-González (2000) | Pest species or outbreak overview | Mexico | No | No | 22.5175, -101.6131 | Country-wide study, used central point for Mexico. |
| Waters (2015) | Farmer perception | Belize | No | No | 17.19167, -88.49889 | Country-wide study. Used approximate central point for Belize, found using Google Earth. |

**References:**

Abba AM, Zufiaurre E, Codesido M, Bilenca DN. 2015. Burrowing activity by armadillos in agroecosystems of central Argentina: biogeography, land use, and rainfall effects. *Agriculture, Ecosystems & Environment* 200:54–61. DOI: 10.1016/j.agee.2014.11.001.

Abrahams MI, Peres CA, Costa HC. 2018. Manioc losses by terrestrial vertebrates in western Brazilian Amazonia. *The Journal of Wildlife Management* 82:734–746. DOI: 10.1002/jwmg.21443.

Aguiar LM, Moro-Rios RF, Silvestre T, Silva-Pereira JE, Bilski DR, Passos FC, Sekiama ML, Rocha VJ. 2011. Diet of brown-nosed coatis and crab-eating raccoons from a mosaic landscape with exotic plantations in southern Brazil. *Studies on Neotropical Fauna and Environment* 46:153–161. DOI: 10.1080/01650521.2011.640567.

Albarracín V, Aliaga-Rossel E. 2018. Bearly guilty: Understanding human–Andean bear conflict regarding crop losses. *Ethnobiology Letters* 9:323–332. DOI: 10.14237/ebl.9.2.2018.1300.

de Almeida Jácomo AT, Furtado MM, Kashivakura CK, Marinho-Filho J, Sollmann R, Tôrres NM, Silveira L. 2013. White-lipped peccary home-range size in a protected area and farmland in the central Brazilian grasslands. *Journal of Mammalogy* 94:137–145. DOI: 10.1644/11-MAMM-A-411.1.

Aris ID, León De Castro MW, Ruesta PV. 2008. Relaciones entre los pobladores rurales y los carnívoros altoandinos del distrito de Anco, centro-Sur del Perú. *Ecología Aplicada* 7:43–48. DOI: 10.21704/rea.v7i1-2.358.

Arroyo-Quiroz I, García-Barrios R, Argueta-Villamar A, Smith R, Pérez-Gil R. 2017. Local perspectives on conflicts with wildlife and their management in the Sierra Gorda Biosphere Reserve, Mexico. *Journal of Ethnobiology*, 37: 719-742. DOI: 10.2993/0278-0771-37.4.719

Avery ML, Tillman EA, Laukert CC. 2001. Evaluation of chemical repellents for reducing crop damage by Dickcissels in Venezuela. *International Journal of Pest Management* 47:311–314. DOI: 10.1080/09670870110065235.

Barceló I, Guzmán-Aranda JC, Chávez-Ramírez F, Powell LA. 2012. Rural Inhabitant Perceptions of Sandhill Cranes in Wintering Areas of Northern Mexico. *Human Dimensions of Wildlife* 17:301–307. DOI: 10.1080/10871209.2012.668610.

Basili GD, Temple SA. 1999a. Dickcissels and crop damage in Venezuela: Defining the problem with ecological models. *Ecological applications* 9:732–739. DOI: 10.1890/1051-0761(1999)009[0732:DACDIV]2.0.CO;2.

Basili GD, Temple SA. 1999b. Winter ecology, behavior, and conservation needs of Dickcissels in Venezuela. *Studies in Avian Biology*. 19:289–299.

Berón IJ, Bortoluzzi A, Dardanelli S. 2020. Avifauna de cuatro plantaciones de higo (*Ficus carica*) en el centro-este de Argentina. *Ornitología neotropical* 31:34–41.

Bou N, Dardanelli S, Olivera L, Tellechea G, Orduna LA, Canavelli S, Rodriguez E. 2016. Desarrollo de un método para evaluar el daño ocasionado por aves en cultivos comerciales de soja recién emergida. *Idesia* 34:67-74. DOI: 10.4067/S0718-34292016005000036.

Boulton AM, Horrocks JA, Baulu J. 1996. The Barbados vervet monkey (*Cercopithecus aethiops sabaeus*): Changes in population size and crop damage, 1980–1994. *International Journal of Primatology* 17:831-844. DOI: 10.1007/BF02735267.

Bourne GR. 1981. Food habits of Black-bellied Whistling Ducks occupying rice culture habitats. *The Wilson Bulletin* 93:551–554.

Bruggers RL, Rodriguez E, Zaccagnini, ME. 1998. Planning for bird pest problem resolution: A case study. *International Biodeterioration & Biodegradation* 42:173–184. DOI: 10.1016/S0964-8305(98)00046-8.

Bucher EH, Aramburú RM. 2014. Land-use changes and monk parakeet expansion in the Pampas grasslands of Argentina. *Journal of Biogeography* 41:1160–1170. DOI: 10.1111/jbi.12282.

Bucher EH, Ranvaud RD. 2006. Eared dove outbreaks in South America: patterns and characteristics. *Acta Zoologica Sinica* 52:564–567.

Calamari NC, Canavelli SB, Cerezo A, Dardanelli S, Bernardos JN, Zaccagnini ME. 2018. Variations in pest bird density in Argentinean agroecosystems in relation to land use and/or cover, vegetation productivity and climate. *Wildlife Research* 45:668–678. DOI: 10.1071/WR17167.

Canavelli SB, Aramburú R, Zaccagnini ME. 2012. Aspectos a considerar para disminuir los conflictos originados por los daños de la cotorra (*Myiopsitta monachus*) en cultivos agrícolas. *Hornero* 27:89-101.

Canavelli SB, Swisher ME, Branch LC. 2013. Factors related to farmers' preferences to decrease monk parakeet damage to crops. *Human Dimensions of Wildlife* 18:124-137. DOI: 10.1080/10871209.2013.745102.

Canavelli SB, Branch LC, Cavallero P, González C, Zaccagnini ME. 2014. Multi-level analysis of bird abundance and damage to crop fields. *Agriculture, Ecosystems and Environment* 197:128–136. DOI: 10.1016/j.agee.2014.07.024.

Can-Hernández G, Villanueva-García C, Gordillo-Chávez EJ, Pacheco-Figueroa CJ, Pérez-Netzahual E, García-Morales R. 2019. Wildlife damage to crops adjacent to a protected area in southeastern Mexico: farmers’ perceptions versus actual impact. *Human-Wildlife Interactions* 13:423–438. DOI: 10.26077/9gqj-5m75.

de Carvalho ALC, Araújo AR, Machado TMM, Ribon R, Lopes LE. 2019. Wildlife and damage to agriculture: an ethnobiological approach with rural producers in southeastern Brazil. *Revista Brasileira de Ornitologia* 27:17–26. DOI: 10.1007/bf03544442.

Castilho LC, de Vleeschouwer KM, Milner-Gulland EJ, Schiavetti A. 2018. Attitudes and behaviors of rural residents toward different motivations for hunting and deforestation in protected areas of the northeastern Atlantic forest, Brazil. *Tropical Conservation Science* 11:1–14. DOI: 10.1177/1940082917753507.

Castillo-Chinchilla M, Piedra-Castro L, Sandoval-Hernández I, Carvajal-Sánchez JP. 2018. Conocimiento popular de los mamíferos del Parque Nacional Barra Honda, Nicoya, Costa Rica. *Uniciencia* 32:82–95. DOI: 10.15359/ru.32-2.6.

Castillo-López IF, Rodríguez-Africano PE, Montes-Pérez RC, González-Valderrama DM. 2017. Fauna silvestre que afecta los cultivos en Boyacá y control del daño a cultivos de maíz. *Ciencia y Agricultura* 14:75–84. DOI: 10.19053/01228420.v14.n1.2017.6090.

Cervo IB, Guadagnin DL. 2020. Wild boar diet and its implications on agriculture and biodiversity in Brazilian forest–grassland ecoregions. *Animal Biodiversity and Conservation* 43:123–136. DOI: 10.32800/abc.2020.43.0123.

Chaves ÓM, Bicca-Marques JC. 2017. Crop feeding by brown howlers (*Alouatta guariba clamitans*) in forest fragments: the conservation value of cultivated species. *International Journal of Primatology* 38:263–281. DOI: 10.1007/s10764-016-9927-8.

Cirne MP, López-Iborra GM. 2005. Breeding biology of Chestnut-capped Blackbirds in rice paddies in southern Brazil. *Journal of Field Ornithology* 76:411–416. DOI: 10.1648/0273-8570-76.4.411.

Codesido M, Zufiaurre E, Bilenca D. 2015. Relationship between pest birds and landscape elements in the Pampas of Central Argentina. *Emu* 115:80–84. DOI: 10.1071/MU13110.

Cornejo BV. 2000. Pocket gopher (*Orthogeomys hispidus hispidus*) damage in sugarcane fields in the state of Veracruz, Mexico. *Proceedings of the Vertebrate Pest Conference* 19:358–361. DOI: 10.5070/V419110222

Corrêa FM, Chaves ÓM, Printes RC, Romanowski HP. 2018. Surviving in the urban–rural interface: Feeding and ranging behavior of brown howlers (*Alouatta guariba clamitans*) in an urban fragment in southern Brazil. *American Journal of Primatology* 80: e22865. DOI: 10.1002/ajp.22865.

Cossios ED, Ridoutt FV, Donoso AL. 2018. Relationships between Molina’s hog nosed skunks, *Conepatus chinga* (Mammalia, Mephitidae) and human beings in the Chaupihuaranga river basin, Pasco, Peru. *Ecología Aplicada* 17:207–214. DOI: 10.21704/rea.v17i2.1240.

Costán AS, Sarasola JH. 2017. Eared dove (*Zenaida auriculata*) granivory and its role in seed dispersal in semiarid forests of central Argentina. *Ornitología Neotropical* 28:43-50.

Dardanelli S, Fandiño B, Calamari NC, Canavelli SB, Zaccagnini ME. 2016. ¿Eligen las palomas y cotorras los lotes de soja (*Glycine max*) en emergencia? Un caso de estudio en agroecosistemas de Entre Ríos, Argentina. *Revista Mexicana de Biodiversidad* 87:1308–1314. DOI: 10.1016/j.rmb.2016.09.006.

Dore KM, Eller AR, Eller JL. 2018. Identity construction and symbolic association in farmer-vervet monkey (*Chlorocebus aethiops sabaeus*) interconnections in St. Kitts. *Folia Primatologica* 89:63–80. DOI: 10.1159/000479064.

Doutel-Ribas C, Martins FI, Campos Z, Piovezan U, Tomas W, Silva VS, Pellegrin A, Mourão G. 2019. Invasive wild boars and native mammals in agroecosystems in the Atlantic Forest of Western Brazil. *Pesquisa Agropecuária Brasileira* 54:e00241. DOI: 10.1590/S1678-3921.PAB2019.V54.00241.

Eiris GC, Barreto GR. 2009. Home range of mars rats, *Holochilus sciureus*, a rodent pest in rice fields of Venezuela. *Interciencia* 34:400–405.

Engeman RM, Laborde JE, Constantin BU, Shwiff SA, Hall P, Duffiney A, Luciano F. 2010. The economic impacts to commercial farms from invasive monkeys in Puerto Rico. *Crop Protection* 29:401–405. DOI: 10.1016/j.cropro.2009.10.021.

Escobar-Lasso S, Cepeda-Duque JC, Gil-Fernández M, González-Maya JF. 2020. Is the banana ripe? Andean bear-human conflict in a protected area of Colombia. *Human-Wildlife Interactions* 14:200–215. DOI: 10.26077/6e5e-089e.

Felix GA, Almeida Paz ICL, Piovezan U, Garcia RG, Lima KAO, Nääs IA, Salgado DD, Pilecco M, Belloni M. 2014. Feeding behavior and crop damage caused by capybaras (*Hydrochoerus hydrochaeris*) in an agricultural landscape. *Brazilian Journal of Biology* 74:779–786. DOI: 10.1590/1519-6984.02113.

Ferraz KMPMB, Lechevalier M-A, Couto HTZ, Verdade LM. 2003. Damage caused by capybaras in a corn field. *Scientia Agricola* 60:191–194. DOI: h10.1590/S0103-90162003000100029

Ferraz KMPM de B, Ferraz SF de B, Moreira JR, Couto HTZ, Verdade LM. 2007. Capybara (*Hydrochoerus* *hydrochaeris*) distribution in agroecosystems: a cross-scale habitat analysis. *Journal of Biogeography* 34:223–230. DOI: 10.1111/j.1365-2699.2006.01568.x.

Ferraz KMPMB, Peterson AT, Scachetti-Pereira R, Vettorazzi CA, Verdade LM. 2009. Distribution of capybaras in an agroecosystem, southeastern Brazil, based on ecological niche modeling. *Journal of Mammalogy* 90:189–194. DOI: 10.1644/07-MAMM-A-338.1.

Figueroa J. 2013. Revisión de la dieta del oso andino *Tremarctos ornatus* (Carnivora: Ursidae) en América del Sur y nuevos registros para el Perú. *Revista del Museo Argentino de Ciencias Naturales* 15: 1–27

Flores-Armillas VH, López-Medellín X, Barrios RG, MacGregor-Fors I, Valenzuela-Galván D. 2020. Landscape features associated with damage to Maize (*Zea mays*) fields in Central México: A comparison of wind and wildlife damage. *Agriculture* 10:460. DOI: 10.3390/agriculture10100460.

de Freitas CH, Setz EZF, Araújo ARB, Gobbi N. 2008. Agricultural crops in the diet of bearded capuchin monkeys, *Cebus libidinosus* Spix (Primates: Cebidae), in forest fragments in southeast Brazil. *Revista Brasileira de Zoologia* 25:32–39. DOI: 10.1590/S0101-81752008000100006.

Fuentes ER, Campusano C. 1985. Pest outbreaks and rainfall in the semi-arid region of Chile. *Journal of Arid Environments* 8:67–72. DOI: 10.1016/s0140-1963(18)31338-7.

Galetti M. 1993. Diet of the scaly-headed parrot (*Pionus maximiliani*) in a semideciduous forest in southeastern Brazil. *Biotropica* 25:419–425. DOI: 10.2307/2388865.

García JM, Peiró V. 2016. Feeding use of a tropical agroecosystem (Cuba) by Mourning dove (*Zenaida macroura* L). *The Journal of Animal and Plant Sciences* 26:1879–1885.

García-Mendoza PJ, Prieto-Rosales GP. 2019. Análisis preliminar de los daños ocasionados al maíz por vertebrados plagas en la localidad Pilcos, Colcabamba, Perú. *Tayacaja* 2:111–126. DOI: 10.46908/rict.v2i1.43.

González DV, Acosta Perez N. 2002. Assessment of bird damage to early-ripening rice in Cuautla, Morelos State, Mexico. *Proceedings of the Vertebrate Pest Conference* 20:157–160. DOI: 10.5070/v420110237.

Gorosábel A, Pedrana J, Bernad L, Caballero VJ, Muñoz SD, Maceira NO. 2019. Evaluating the impacts and benefits of sheldgeese on crop yields in the Pampas region of Argentina: A contribution for mitigating the conflicts with agriculture. *Agriculture,* *Ecosystems and Environment* 279:33–42. DOI: 10.1016/j.agee.2019.04.002.

Hilje L. 1992. Daño y combate de los roedores plaga en Costa Rica. *Manejo Integrado de Plagas y Agroecología* 23:32–38.

Horrocks JA, Baulu J. 1988. Effects of trapping on the vervet (*Cercopithecus aethiops sabaeus*) population in Barbados. *American Journal of Primatology* 15:223–233. DOI: 10.1002/ajp.1350150305.

Horrocks J, Baulu J. 1994. Food competition between vervets (*Cercopithecus aethiops sabaeus*) and farmers in Barbados: implications for management. *Revue d'Ecologie* 49:281–294.

Horváth A, March IJ, Wolf JHD. 2001. Rodent diversity and land use in Montebello, Chiapas, Mexico. *Studies on Neotropical Fauna and Environment* 36:169–176. DOI: 10.1076/snfe.36.3.169.2130.

Ibañez LM, Andreucci F, Montalti D. 2016. Primer registro de daño a cultivo de frutales por el estornino pinto (*Sturnus vulgaris*) (Passeriformes: Sturnidae) en Argentina. *Acta Zoológica Lilloana*. 60:177–180.

Jackson JE. 1988. Terrestrial mammalian pests in Argentina-an overview. *Proceedings of the Vertebrate Pest Conference* 13:196–198.

Key GE, de la Piedra Constantino R. 1992. The field rat control campaign, Chiapas, Mexico. *Proceedings of the Vertebrate Pest Conference* 15:268–271.

Lima M, Peres CA, Abrahams MI, Junior CAS, Costa GM, Santos RC. 2019. The paradoxical situation of the white-lipped peccary (*Tayassu pecari*) in the state of Mato Grosso, Brazil. *Perspectives in Ecology and Conservation* 17:36–39. DOI: 10.1016/j.pecon.2018.12.001.

Lins PGAS, Ferreira RG. 2019. Competition during sugarcane crop raiding by blond capuchin monkeys (*Sapajus flavius*). *Primates* 60:81–91. DOI: 10.1007/s10329-018-0698-z.

Lobão ESP, Nogueira-Filho SLG. 2011. Human-wildlife conflicts in the Brazilian Atlantic Forest. *Suiform Soundings* 10:14–22.

López-Torres AL, Claudio-Hernández HJ, Rodríguez-Gómez CA, Longo AV, Joglar RL. 2012. Green Iguanas (*Iguana iguana*) in Puerto Rico: Is it time for management? *Biological Invasions* 14:35–45. DOI: 10.1007/s10530-011-0057-0.

Loza-del-Carpio A, Clavitea J, Delgado P. 2016. Incidencia de aves granívoras y su importancia como plagas en el cultivo de quinua (*Chenopodium quinoa* Willd.) en el altiplano Peruano. *Bioagro* 28:139–150.

MacGregor-Fors I, Calderón-Parra R, Meléndez-Herrada A, López-López S, Schondube JE. 2011. Pretty, but dangerous! Records of Monk Parakeets (*Myiopsitta monachus*) in Mexico and their possible invasion effects. *Revista Mexicana de Biodiversidad* 82:1053–1056. DOI: 10.22201/ib.20078706e.2011.3.721.

Marchand G. 2016. Analyse de la dimension spatiale des conflits homme/faune sauvage dans la réserve de développement durable de la rivière Uatumã (Amazonas, Brésil). *Cybergeo: European Journal of Geography* (online). DOI: 10.4000/cybergeo.27807.

McKinney T. 2011. The effects of provisioning and crop-raiding on the diet and foraging activities of human-commensal white-faced Capuchins (*Cebus capucinus*). *American Journal of Primatology* 73:439–448. DOI: 10.1002/ajp.20919.

McKinney T. 2019. Ecological and behavioural flexibility of mantled howlers (*Alouatta palliata*) in response to anthropogenic habitat disturbance. *Folia Primatologica* 90:456–469. DOI: 10.1159/000499825.

Melo C, Cheschini J. 2012. Daños causados por las aves en sorgo (*Sorghum bicolor*) en Brasil central. *Bioagro* 24:33–38.

Mendonça LET, Souto CM, Andrelino, LL, Souto WMS, Vieira WLS, Alves RRN. 2012. Conflitos entre pessoas e animais silvestres no Semiárido paraibano e suas implicações para conservação. *Sitientibus série Ciências Biológicas* 11:185–199. DOI: 10.13102/scb107.

Mitchell B, Bruggers RL. 1985. Aspects of woodpecker damage to cacao in the Dominican Republic. *Tropical Pest Management* 31:148–152. DOI: 10.1080/09670878509370969.

Monge J. 1999. Impacto potencial de la taltuza (*Orthogeomys* sp.) en el cultivo del pejibaye (*Bactris gasipaes*) en Costa Rica. *Agronomía Mesoamericana* 10:133–136. DOI: 10.15517/am.v10i2.17989.

Monge J. 2013. Lista actualizada de aves dañinas en Costa Rica (2012). *UNED Research Journal* 5:111–120. DOI: 10.22458/urj.v5i1.197.

Monge-Meza J, Linares-Orozco J. 2010. Presencia del zorro de cuatro ojos (*Philander opossum*) en el cultivo de piña (*Ananas comusus*). *Agronomía Mesoamericana* 21:343–347. DOI: 10.15517/am.v21i2.4898.

Monge-Meza J. 2011. El impacto de las taltuzas en el cultivo de banano. *Agronomía Mesoamericana* 22:167–174.

Monge-Meza J, Herrera-Murillo F, Arias-Reverón J. 2014. Daños de la rata *Sigmodon hirsutus* (Rodentia: Cricetidae) al cultivo de maní (*Arachis hypogaea*) en Alajuela, Costa Rica. *UNED Research Journal* 6:81–86. DOI: 10.22458/urj.v6i1.318

Naughton-Treves L, Mena JL, Treves A, Alvarez N, Radeloff VC. 2003. Wildlife survival beyond park boundaries: the impact of slash-and-burn agriculture and hunting on mammals in Tambopata, Peru. *Conservation Biology* 17:1106–1117. DOI: 10.1046/j.1523-1739.2003.02045.x

Olivera L, Rodríquez E, Ceretta S, Beyhaut E. 2016. Repelentes de aves aplicados a la semilla de soja: compatibilidad con el inoculante y residualidad en cotiledones. *Agrociencia* 20:51–60.

Parra JG, García AA, Poleo CJ, Fuentes LM. 2012. Aspectos reproductivos y daños causados por una comunidad de roedores en arroz bajo siembra directa en el sistema de riego río Guárico. *Agronomía Tropical* 62:163–170.

Pedrana J, Bernad L, Maceira NO, Isacch JP. 2014. Human-Sheldgeese conflict in agricultural landscapes: Effects of environmental and anthropogenic predictors on Sheldgeese distribution in the southern Pampa, Argentina. *Agriculture, Ecosystems and Environment* 183:31–39. DOI: 10.1016/j.agee.2013.09.029.

Pedrosa F, Salerno R, Padilha FVB, Galetti M. 2015. Current distribution of invasive feral pigs in Brazil: Economic impacts and ecological uncertainty. *Natureza e Conservação* 13:84–87. DOI: 10.1016/j.ncon.2015.04.005.

Pereira CZ, Rosa C, Zanzini ACS. 2019. Perception of presence, impact and control of the invasive species *Sus scrofa* in the local community living near the Itatiaia National Park, Brazil. *Ethnobiology and Conservation* 8:1–11. DOI: 10.15451/ec2019-06-8.06-1-11.

Pérez EM, Bulla L. 2000. Dietary relationships among four granivorous doves in Venezuelan savannas. *Journal of Tropical Ecology* 16:865–882. DOI: 10.1017/S0266467400001772.

Pérez E, Pacheco LF. 2006. Damage by large mammals to subsistence crops within a protected area in a montane forest of Bolivia. *Crop Protection* 25:933–939. DOI: 10.1016/j.cropro.2005.12.005.

Pérez E, Pacheco LF. 2014. Mitigación de daños provocados por fauna silvestre en cultivos agrícolas en un bosque montano de Bolivia. *Revista de Biología Tropical* 62:1495–1507.

Peyton B. 1980. Ecology, distribution, and food habits of spectacled bears, *Tremarctos ornatus*, in Peru. *Journal of Mammalogy* 61:639–652. DOI: 10.2307/1380309.

Poleo CJ, Fuentes L, Vivas L. 2010. Caracterización reproductiva de una población de *Zygodontomys brevicauda* (Rodentia: Cricetidae) capturada en siembras de arroz del estado Guárico, Venezuela. *Agronomía Tropical* 60:43–48.

Ranvaud R, de Freitas KC, Bucher EH, Dias HS, Avanzo VC, Alberts CC. 2001. Diet of Eared Doves (*Zenaida auriculata*, Aves, Columbidae) in a sugar-cane colony in South-eastern Brazil. *Brazilian Journal of Biology* 61:651–660. DOI: 10.1590/S1519-69842001000400015

Renfrew RB, Saavedra AM. 2007. Ecology and conservation of Bobolinks (*Dolichonyx oryzivorus*) in rice production regions of Bolivia. *Ornitología Neotropical* 18:61–74*.*

Renfrew RB, Hill JM, Kim DH, Romanek C, Perlut NG. 2017. Winter diet of Bobolink, a long-distance migratory grassland bird, inferred from feather isotopes. *The Condor* 119:439–448. DOI: 10.1650/CONDOR-16-162.1.

Robles J, Jacobsen SE, Rasmussen C, Otazu V, Mandujano J. 2003. Plagas de aves en Quinua (*Chenopodium quinoa* Willd.) y medidas de control en el Perú central. *Revista Peruana de Entomología*. 43:147–151.

Rocha LC, Fortes VB. 2015. Perceptions and attitudes of rural residents towards capuchin monkeys, in the area of influence of the Dona Francisca hydroelectric power plant, South Brazil. *Ambiente e Sociedade* 18:19–34. DOI: 10.1590/1809-4422ASOC825V1842015

Rodriguez EN, Avery ML. 1996. *Agelaius* blackbirds and rice in Uruguay and the southeastern United States. *Proceedings of the Seventeenth Vertebrate Pest Conference* 17:94–98.

Rodriguez EN, Bruggers RL, Bullard RW, Cook R. 1995. An integrated strategy to decrease eared dove damage in sunflower crops. *National Wildlife Research Center Repellents Conference* 409–421.

Rodriguez EN, Tiscornia G, Tobin ME. 2004. Bird depredations in Uruguayan vineyards. *Proceedings of the Vertebrate Pest Conference* 21:136–139.

Romero-Balderas KG, Naranjo EJ, Morales HH, Nigh RB. 2006. Daños ocasionados por vertebrados silvestres al cultivo de maíz en la Selva Lacandona, Chiapas, México. *Interciencia* 31:276–283.

Rosa CA, Wallau MO, Pedrosa F. 2018. Hunting as the main technique used to control wild pigs in Brazil. *Wildlife Society Bulletin* 42:111–118. DOI: 10.1002/wsb.851.

Sánchez R, Ballari SA, Bucher EH, Masello JF. 2016. Foraging by burrowing parrots has little impact on agricultural crops in northeastern Patagonia, Argentina. *International Journal of Pest Management* 62:326–335. DOI: 10.1080/09670874.2016.1198061.

Sánchez-Cordero V, Martínez-Meyer E. 2000. Museum specimen data predict crop damage by tropical rodents. *Proceedings of the National Academy of Sciences* 97:7074–7077. DOI: 10.1073/pnas.97.13.7074

Santos GC. 2018. Characteristics of rodent outbreaks in the low San Francisco Sergipano (Sergipe, Brazil) and influence of anomalies on sea surface temperature on temperatures in this region. *International Journal of Design and Nature and Ecodynamics* 13:156–165. DOI: 10.2495/DNE-V13-N2-156-165.

Saucedo Castillo OM, Fernández Pérez LE, Quiñones Ramos R, Rodríguez Valdés G, Moya Álvarez A. 2017. Las aves granívoras y el cultivo del sorgo en la provincia de Villa Clara, Cuba. *Revista Centro Agrícola* 44:36–43.

Silva-Andrade HL, de Andrade LP, Muniz LS, Telino-Júnior WR, Albuquerque UP, Lyra-Neves RM. 2016. Do farmers using conventional and non-conventional systems of agriculture have different perceptions of the diversity of wild birds? Implications for conservation. *PLoS ONE* 11:e0156307. DOI: 10.1371/journal.pone.0156307.

Silva-Rodríguez EA, Ortega-Solís GR, Jiménez JE. 2006. Aves silvestres: actitudes, prácticas y mitos en una localidad rural del sur de Chile. *Boletín Chileno de Ornitología* 12:2–14.

Spagnoletti N, Cardoso TCM, Fragaszy D, Izar P. 2017. Coexistence between humans and capuchins (*Sapajus libidinosus*): comparing observational data with farmers’ perceptions of crop losses. *International Journal of Primatology* 38:243–262. DOI: 10.1007/s10764-016-9926-9.

Trivedi MR, Cornejo FH, Watkinson AR. 2004. Seed predation on brazil nuts (*Bertholletia excelsa*) by macaws (Psittacidae) in Madre de Dios, Peru. *Biotropica* 36:118–122. DOI: 10.1646/03050.

Valencia D. 1980. Rat control in coconut palms in Colombia. *Proceedings of the Vertebrate Pest Conference* 9:110–113.

Valencia D, Elias DJ, Ospina JA. 1994. Rodent pests in Colombian agriculture*. Proceedings of the Vertebrate Pest Conference* 16: 92–94.

Villa CB, Lopez-Forment W, Villa CM, Prescott CV. 1998. Not all sigmodontine rodents in the sugarcane fields in coastal Veracruz, Mexico, are pests. *Proceedings of the Vertebrate Pest Conference* 18:236–241. DOI: 10.5070/V418110096.

Villafaña FM, Pupo MS, Blanco JR, Sánchez Rojas LG, Campos Muñoz C. 1999. Evaluación del impacto del biorrodenticida Biorat en poblaciones de roedores establecidos en varios cultivos en la República de Costa Rica. *Revista Cubana de Medicina Tropical* 51:185–188.

del Villar-González D. 2000. Principales vertebrados plaga en México: situación actual y alternativas para su manejo. *Revista Chapingo Serie Ciencias Forestales y del Ambiente* 6:41–54.

Waters S. 2015. Crop-raiding Baird’s tapir provoke diverse reactions from subsistence farmers in Belize. *Tapir Conservation* 24:8–10. DOI: 10.5281/zenodo.22642.
