# Supplementary material for: Crop damage by vertebrates in Latin America: current knowledge and potential future management directions
Source: PeerJ. 2022 Mar 25;10:e13185. doi: 10.7717/peerj.13185 (PMC8958972; doi:10.7717/peerj.13185)
Supplement: Supplemental Information 2 — Including information on the number of studies they appear on, the crop genera they interact with and the protection techniques that have been used on them. [file peerj-10-13185-s002.docx]

**Crop damage by vertebrates in Latin America: current knowledge and potential future management directions**

**Adrián Alejandro Cuesta Hermira, Fernanda Michalski**

Supplementary Material

**Table S2: List of vertebrate species reported to produce crop damages across the 113 reviewed studies.**

Including information on the number of studies they appear on, the crop genera they interact with and the protection techniques that have been used on them.

| **Class/Order** | **Vertebrate taxon** | **Number of studies** | **Crop genera** | **Protection techniques** | | |  |
| --- | --- | --- | --- | --- | --- | --- | --- |
|  |  |  |  | **Effective** | **Not effective** | **Undetermined** |  |
| Mammals / Artiodactyla | *Mazama americana* | 1 | *Manihot* | - | - | Hunting (Weapons, Dogs, Traps), Vigilance (People), Visual deterrents (Scarecrows), Agricultural practices (Field clearing, Firebreaks), Physical barriers (Netting), Acoustic deterrents (Yelling) | |
| Mammals / Artiodactyla | *Mazama* sp. | 1 | *-* | - | Agricultural practices (Field clearing), Physical barriers (Fencing) | - | |
| Mammals / Artiodactyla | *Odocoileus virginianus* | 4 | *Zea*, *Phaseolus*, *Cucurbita*, *Cicer* | - | - | Hunting (Weapons), Poisoning, Chemical repellents (Soap), Visual deterrents (Reflective objects, Scarecrows), Acoustic deterrents (Firecrackers), Vigilance (People, Guard dogs) | |
| Mammals / Artiodactyla | *Pecari tajacu* | 9 | *Bactris, Colocasia, Manihot, Musa, Phaseolus, Xanthosoma, Zea* | Physical barriers (Wire mesh exclosures), Hunting, Agricultural practices (Field clearing), Olfactory deterrents (Human odors), Visual deterrents (Flags), Vigilance (People) | Agricultural practices (Field clearing), Physical barriers (Fencing) | Acoustic deterrents (Firecrackers, Firearms, Yelling), Hunting (Undetermined, Weapons, Dogs, Traps), Vigilance (People), Agricultural practices (Field clearing, Firebreaks), Physical barriers (Fencing, Netting), Visual deterrents (Scarecrows). | |
| Mammals / Artiodactyla | *Sus scrofa* | 7 | *Avena, Cucurbita, Daucus, Fragaria, Glycine, Lolium, Oryza, Saccharum, Sorghum, Zea*, *Manihot* | - | Acoustic deterrents (Firecrackers, Gas cannon), Visual deterrents (Scarecrows, Reflective objects), Vigilance (People, Guard dogs), Physical barriers (Fencing, Netting), Chemical repellents, Hunting, Agricultural practices (Providing alternative food sources) | Hunting (Undetermined, Dogs, Weapons, Traps) | |
| Mammals / Artiodactyla | *Tayassu pecari* | 3 | *Glycine, Zea*, *Sorghum*, *Panicum* | Hunting (Weapons, Dogs, Traps), Poisoning (Carbofuran) | Physical barriers (Electric fencing, Trenches), Agricultural practices (Providing alternative food sources, Barrier crops), Acoustic deterrents (Firecrackers) | - | |
| Mammals / Artiodactyla | *Tayassu* sp. | 1 | *-* | - | - | Hunting | |
| Mammals / Carnivora | *Cerdocyon thous* | 1 | *Zea* | - | - | Hunting | |
| Mammals / Carnivora | *Conepatus chinga* | 3 | *Zea* | - | - | Hunting (Weapons, Traps), Acoustic deterrents (Fireworks), Vigilance (People) | |
| Mammals / Carnivora | *Eira barbara* | 1 | *-* | - | - | Hunting | |
| Mammals / Carnivora | *Mustela frenata* | 1 | *-* | - | - | - | |
| Mammals / Carnivora | *Nasua narica* | 6 | *Zea*, *Phaseolus*, *Arachis*, *Carica*, *Persea*, *Mangifera*, *Musa* | - | - | Hunting (Undetermined, Weapons, Traps), Poisoning, Chemical repellents (Soap), Visual deterrents (Reflective objects, Scarecrows), Acoustic deterrents (Firecrackers, Firearms), Vigilance (People, Guard dogs) | |
| Mammals / Carnivora | *Nasua nasua* | 5 | *Carica, Colocasia, Manihot, Musa, Zea, Citrullus, Persea, Morus, Eriobotrya, Vassobia* | Physical barriers (Wire mesh exclosures), Hunting, Agricultural practices (Field clearing), Olfactory deterrents (Human odors), Visual deterrents (Flags), Vigilance (People) | Acoustic deterrents (Firecrackers, Gas cannon), Visual deterrents (Scarecrows, Reflective objects), Vigilance (People), Physical barriers (Fencing, Netting), Chemical repellents, Hunting, Agricultural practices (Providing alternative food sources) | - | |
| Mammals / Carnivora | *Procyon cancrivorus* | 2 | *Musa, Eriobotrya* | - | - | Hunting (Undetermined, Traps), Acoustic deterrents (firecrackers, firearms) | |
| Mammals / Carnivora | *Procyon lotor* | 5 | *Zea* | - | - | Hunting (Undetermined, Weapons), Poisoning, Chemical repellents (Soap), Visual deterrents (Reflective objects, Scarecrows), Acoustic deterrents (Firecrackers), Vigilance (People, Guard dogs) | |
| Mammals / Carnivora | Procyonidae | 1 | *Zea* | - | - | Hunting (Weapons), Poisoning, Chemical repellent (Soap), Visual deterrents (Reflective objects, Scarecrows), Acoustic deterrents (Firecrackers), Vigilance (People, Guard dogs) | |
| Mammals / Carnivora | *Tremarctos ornatus* | 4 | *Annona, Ananas, Opuntia, Cucurbita, Manihot, Persea, Psidium, Musa, Saccharum, Zea, Citrus, Lucuma, Solanum* | - | Physical barriers (Fencing) | Hunting, Acoustic deterrents (Fireworks), Vigilance (People), Agricultural practices (Field clearing), Visual deterrents (Fire), Olfactory deterrents (Burnt rubber) | |
| Mammals / Carnivora | *Urocyon cinereoargenteus* | 1 | *Zea* | - | - | - | |
| Mammals / Chiroptera | Chiroptera | 1 | *Zea* | - | Palatable deterrents (Chile), Olfactory deterrents (Creolina) | - | |
| Mammals / Cingulata | *Cabassous unicinctus* | 1 | *Manihot, Phaseolus, Theobroma* | - | - | Hunting (Undetermined, Traps), Acoustic deterrents (firecrackers, firearms) | |
| Mammals / Cingulata | *Chaetophractus* sp. | 1 | - | - | - | - | |
| Mammals / Cingulata | *Chaetophractus villosus* | 1 | *Glycine, Helianthus, Triticum, Zea* | - | - | - | |
| Mammals / Cingulata | Dasypodidae | 1 | *Zea* | - | Acoustic deterrents (Firecrackers, Gas cannon), Visual deterrents (Scarecrows, Reflective objects), Vigilance (People), Physical barriers (Fencing, Netting), Chemical repellents, Hunting, Agricultural practices (Providing alternative food sources) | - | |
| Mammals / Cingulata | *Dasypus hybridus* | 1 | *Glycine, Helianthus, Triticum, Zea* | - | - | - | |
| Mammals / Cingulata | *Dasypus novemcinctus* | 2 | *Manihot, Phaseolus, Theobroma*, *Cicer*, *Pisum* | - | - | Hunting (Undetermined, Traps), Acoustic deterrents (firecrackers, firearms) | |
| Mammals / Cingulata | *Dasypus* sp. | 1 | *-* | - | - | - | |
| Mammals / Cingulata | *Euphractus sexcinctus* | 1 | *Manihot, Phaseolus, Theobroma* | - | - | Hunting (Undetermined, Traps), Acoustic deterrents (firecrackers, firearms) | |
| Mammals / Didelphimorphia | *Didelphis aurita* | 1 | *Elaeis* | - | - | Hunting (Undetermined, Traps), Acoustic deterrents (firecrackers, firearms) | |
| Mammals / Didelphimorphia | *Didelphis marsupialis* | 1 | *Zea* | - | Palatable deterrents (Chile), Olfactory deterrents (Creolina) | - | |
| Mammals / Didelphimorphia | *Didelphis* sp. | 2 | *Manihot* | Hunting, Agricultural practices (Field clearing), Olfactory deterrents (Human odours), Visual deterrents (Flags), Vigilance (People) | Acoustic deterrents (Firecrackers, Gas cannon), Visual deterrents (Scarecrows, Reflective objects), Vigilance (People), Physical barriers (Fencing, Netting), Chemical repellents, Hunting, Agricultural practices (Providing alternative food sources) | - | |
| Mammals / Didelphimorphia | *Didelphis virginiana* | 1 | *Zea* | - | - | - | |
| Mammals / Didelphimorphia | *Metachirus nudicaudatus* | 1 | *Theobroma* | - | - | Hunting (Undetermined, Traps), Acoustic deterrents (firecrackers, firearms) | |
| Mammals / Didelphimorphia | *Philander opossum* | 1 | *Arachis* | - | - | - | |
| Mammals / Lagomorpha | Leporidae | 2 | *Zea*, *Phaseolus* | - | Palatable deterrents (Chile), Olfactory deterrents (Creolina) | - | |
| Mammals / Lagomorpha | *Lepus* *europaeus* | 2 | *-* | - | - | Poisonig | |
| Mammals / Lagomorpha | *Lepus* sp. | 1 | *Capsicum* | - | Poisoning (Zinc phosphide, Organophosphates) | - | |
| Mammals / Lagomorpha | *Sylvilagus floridanus* | 1 | *-* | - | - | - | |
| Mammals / Perissodactyla | *Tapirus bairdii* | 1 | *Arachis, Brassica, Citrullus, Dioscorea, Phaseolus, Solanum, Zea* | - | - | Hunting | |
| Mammals / Perissodactyla | *Tapirus terrestris* | 1 | *-* | - | - | Hunting | |
| Mammals / Primates | *Alouatta guariba* | 2 | *Araucaria, Citrus, Diospyros, Eriobotrya, Psidium*, *Morus*, *Syzygium*, *Hovenia*, *Melia*, *Ligustrum* | - | - | - | |
| Mammals / Primates | *Alouatta palliata* | 2 | *Mangifera* | - | - | - | |
| Mammals / Primates | *Allouatta* sp. | 1 | *Zea* | - | Acoustic deterrents (Firecrackers, Gas cannon), Visual deterrents (Scarecrows, Reflective objects), Vigilance (People), Physical barriers (Fencing, Netting), Chemical repellents, Hunting, Agricultural practices (Providing alternative food sources) | - | |
| Mammals / Primates | *Cebus capucinus* | 2 | *Cocos, Elaeis*, *Musa* | - | - | - | |
| Mammals / Primates | *Chlorocebus aethiops* | 4 | *Annona*, *Mangifera*, *Spondias*, *Carica*, *Psidium*, *Arachis*, *Passiflora*, *Malus*, *Pisum*, *Musa*, *Prunus*, *Zea*, *Cucumis*, *Blighia*, *Manihot*, *Persea*, *Daucus*, *Ipomoea*, *Cucurbita*, *Solanum*, *Artocarpus*, *Phaseolus*, *Abelmoschus*, *Dioscorea*, *Citrus*, *Colocasia*, *Brassica*, *Allium*, *Beta*, *Saccharum* | - | Hunting (Firearms, Traps) | Vigilance (Dogs), Hunting (Traps), Agricultural practices (Kind of crops, Location of crops, Alternative food sources, Field clearing) | |
| Mammals / Primates | *Erythrocebus patas* | 1 | *Cucurbita*, *Citrullus*, *Cucumis*, *Carica*, *Musa*, *Zea* | - | - | Agricultural practices (Kind of crops) | |
| Mammals / Primates | *Leontopithecus chrysomelas* | 1 | *Musa* | - | - | Hunting (Undetermined, Traps), Acoustic deterrents (firecrackers, firearms) | |
| Mammals / Primates | *Macaca mulatta* | 1 | *Cucurbita*, *Citrullus*, *Cucumis*, *Carica*, *Musa*, *Zea* | - | - | Agricultural practices (Kind of crops) | |
| Mammals / Primates | *Sapajus apella* | 2 | *Zea* | Physical barriers (Wire mesh exclosures), Hunting, Agricultural practices (Field clearing), Olfactory deterrents (Human odors), Visual deterrents (Flags), Vigilance (People) | - | - | |
| Mammals / Primates | *Sapajus flavius* | 1 | *Saccharum* | - | - | - | |
| Mammals / Primates | *Sapajus libidinosus* | 2 | *Sacharum, Ananas, Carica, Mangifera,* *Citrullus, Manihot, Musa, Oryza, Phaseolus, Zea* | Vigilance (People, Guard dogs), Acoustic deterrents (Yelling, Firearms), Visual deterrents (Scarecrows, Fire), Agricultural practices (Early planting) | - | - | |
| Mammals / Primates | *Sapajus nigritus* | 1 | *Zea* | Agricultural practices (Early planting, Crop location) | Vigilance (Guard dogs), Acoustic deterrents | - | |
| Mammals / Rodentia | Rodentia | 1 | *Avena, Coffea, Oryza, Phaseolus, Saccharum, Sorghum, Tritichum, Zea* | Hunting, Agricultural practices (Field clearing), Olfactory deterrents (Human odors), Visual deterrents (Flags), Vigilance (People) | - | - | |
| Mammals / Rodentia | *Abrothrix olivacea* | 1 | *-* | - | - | Poisoning | |
| Mammals / Rodentia | *Akodon azarae* | 1 | *Zea, Panicum, Sorghum, Helianthus* | - | - | - | |
| Mammals / Rodentia | *Callistomys pictus* | 1 | *Musa* | - | - | Hunting (Undetermined, Traps), Acoustic deterrents (firecrackers, firearms) | |
| Mammals / Rodentia | *Calomys laucha* | 1 | *Zea, Panicum, Sorghum, Helianthus* | - | - | - | |
| Mammals / Rodentia | *Calomys musculinus* | 1 | *Zea, Panicum, Sorghum, Helianthus* | - | - | - | |
| Mammals / Rodentia | *Cavia aperea* | 1 | *Oryza* | - | - | - | |
| Mammals / Rodentia | *Chilomys instans* | 1 | *Zea* | - | Palatable deterrents (Chile), Olfactory deterrents (Creolina) | - | |
| Mammals / Rodentia | *Cratogeomys fumosus* | 1 | *Zea, Avena, Phaseolus, Citrullus, Malus, Brassica, Solanum, Allium, Medicago* | - | Poisoning (Zinc phosphide, Aluminum phosphate, Thiodicarb), Hunting (Firearms) | - | |
| Mammals / Rodentia | *Ctenomys* sp. | 1 | *Citrus, Saccharum* | - | - | - | |
| Mammals / Rodentia | *Cuniculus paca* | 7 | *Colocasia, Manihot, Theobroma, Xanthosoma, Zea* | Physical barriers (Wire mesh exclosures), Hunting, Agricultural practices (Field clearing), Olfactory deterrents (Human odors), Visual deterrents (Flags), Vigilance (People) | - | Hunting (Undetermined, Weapons, Traps, Dogs), Poisoning, Chemical repellents (Soap), Visual deterrents (Reflective objects, Scarecrows), Acoustic deterrents (Firecrackers, Firearms, Yelling), Vigilance (People, Guard dogs), Agricultural practices (Field clearing, Firebreaks), Physical barriers (Netting) | |
| Mammals / Rodentia | *Dasyprocta aguti* | 1 | *Manihot* | - | - | Hunting (Undetermined, Traps), Acoustic deterrents (firecrackers, firearms) | |
| Mammals / Rodentia | *Dasyprocta fuliginosa* | 1 | *Manihot* | - | - | Hunting (Weapons, Dogs, Traps), Vigilance (People), Visual deterrents (Scarecrows), Agricultural practices (Field clearing, Firebreaks), Physical barriers (Netting), Acoustic deterrents (Yelling) | |
| Mammals / Rodentia | *Dasyprocta punctata* | 1 | *Manihot, Xanthosoma, Zea* | Hunting, Agricultural practices (Field clearing), Olfactory deterrents (Human odors), Visual deterrents (Flags), Vigilance (People) | - | - | |
| Mammals / Rodentia | *Dasyprocta* sp. | 1 | - | - | Agricultural practices (Field clearing), Physical barriers (Fencing) | - | |
| Mammals / Rodentia | *Dasyprocta variegata* | 2 | *Colocasia, Manihot, Xanthosoma, Zea* | Physical barriers (Wire mesh exclosures) | - | Hunting | |
| Mammals / Rodentia | *Dinomys branickii* | 1 | *Xanthosoma, Zea* | Hunting, Agricultural practices (Field clearing), Olfactory deterrents (Human odors), Visual deterrents (Flags), Vigilance (People) | - | - | |
| Mammals / Rodentia | Echimyidae | 1 | *Manihot* | - | - | Hunting (weapons, dogs, traps), Vigilance (People), Visual deterrents (Scarecrows), Agricultural practices (Field clearing, Firebreaks), Physical barriers (Netting), Acoustic deterrents (Yelling) | |
| Mammals / Rodentia | *Galea musteloides* | 1 | *-* | - | - | - | |
| Mammals / Rodentia | *Handleyomys chapmani* | 1 | *Saccharum* | - | - | - | |
| Mammals / Rodentia | *Holochilus brasiliensis* | 2 | *Oryza, Saccharum* | - | - | - | |
| Mammals / Rodentia | *Holochilus sciureus* | 3 | *Oryza* | - | - | Hunting (dogs), Poisoning (Rodenticides, Organo-phosphide insecticides) | |
| Mammals / Rodentia | *Hydrochoerus hydrochaeris* | 9 | *Bactris, Glycine, Manihot, Oryza, Phaseolus, Saccharum, Zea* | - | Acoustic deterrents (Firecrackers, Gas cannon), Visual deterrents (Scarecrows, Reflective objects), Vigilance (People), Physical barriers (Fencing, Netting), Chemical repellents, Hunting, Agricultural practices (Providing alternative food sources, Field clearing) | Hunting (Undetermined, Traps), Acoustic deterrents (firecrackers, firearms) | |
| Mammals / Rodentia | *Liomys irroratus* | 1 | Saccharum, Zea, Phaseolus | - | Poisoning (Zinc phosphide, Aluminum phosphate, Thiodicarb) | - | |
| Mammals / Rodentia | *Microtus mexicanus* | 2 | Avena, Phaseolus, Medicago, Zea, Triticum, Sorghum, Cucumis, Malus | - | Poisoning (Zinc phosphide, Aluminum phosphate, Thiodicarb) | - | |
| Mammals / Rodentia | Muridae | 2 | *Colocasia, Xanthosoma, Zea* | Physical barriers (Wire mesh exclosures) | - | - | |
| Mammals / Rodentia | *Mus musculus* | 2 | *Capsicum, Saccharum, Citrullus, Zea, Sorghum, Oryza, Phaseolus* | - | Poisoning (Zinc phosphide, Aluminum phosphate, Thiodicarb) | Poisoning | |
| Mammals / Rodentia | *Nectomys squamipes* | 1 | *Theobroma* | - | - | Hunting (Undetermined, Traps), Acoustic deterrents (firecrackers, firearms) | |
| Mammals / Rodentia | *Notocitellus adocetus* | 1 | Zea, Cocos | - | Poisoning (Zinc phosphide, Aluminum phosphate, Thiodicarb), Hunting (Traps) | - | |
| Mammals / Rodentia | *Octodon degus* | 1 | - | - | - | Poisoning | |
| Mammals / Rodentia | *Oligoryzomys fulvescens* | 1 | - | - | - | - | |
| Mammals / Rodentia | *Oligoryzomys longicaudatus* | 1 | *-* | - | - | Poisoning | |
| Mammals / Rodentia | *Oligoryzomys nigripes* | 1 | *Zea, Panicum, Sorghum, Helianthus* | - | - | - | |
| Mammals / Rodentia | *Oligoryzomys* sp. | 1 | *Oryza* | - | - | - | |
| Mammals / Rodentia | *Orthogeomys cavator* | 3 | *Musa*, *Bactris* | Hunting (Traps) | Biological control (Infectious disease, Introduction of predators), Poisoning (Estricnina, Methyl bromide, Metomil, Aluminium phosphate) | Poisoning (Metomil), Hunting (Firearms, Traps), Biological control (Attracting predators) | |
| Mammals / Rodentia | *Orthogeomys cherriei* | 3 | *Manihot*, *Musa*, *Bactris*, *Colocasia*, *Xanthosoma*, *Zea*, *Saccharum*, *Phaseolus*, *Theobroma*, *Coffea,* *Oryza* | Hunting (Traps) | Biological control (Infectious disease, Introduction of predators), Poisoning (Estricnina, Methyl bromide, Metomil, Aluminium phosphate) | Poisoning (Metomil), Hunting (Firearms, Traps), Biological control (Attracting predators) | |
| Mammals / Rodentia | *Orthogeomys heterodus* | 3 | *Musa*, *Bactris*, *Daucus*, *Allium*, *Solanum*, *Zea*, *Pisum*, *Brassica*, *Avena*, *Persea* | Hunting (Traps) | Biological control (Infectious disease, Introduction of predators), Poisoning (Estricnina, Methyl bromide, Metomil, Aluminium phosphate) | Poisoning (Metomil), Hunting (Firearms, Traps), Biological control (Attracting predators) | |
| Mammals / Rodentia | *Orthogeomys hispidus* | 3 | *Zea*, *Saccharum* | Poisoning (Sodium monofuroacetate) | - | - | |
| Mammals / Rodentia | *Orthogeomys* sp. | 1 | *Coffea* | - | - | Poisoning (Zinc phosphide and Diphacinone) | |
| Mammals / Rodentia | *Orthogeomys underwoodi* | 3 | *Musa*, *Bactris*, *Tamarindus* | Hunting (Traps) | Biological control (Infectious disease, Introduction of predators), Poisoning (Estricnina, Methyl bromide, Metomil, Aluminium phosphate) | Poisoning (Metomil), Hunting (Firearms, Traps), Biological control (Attracting predators) | |
| Mammals / Rodentia | *Oryzomys couesi* | 3 | *Saccharum,* *Oryza, Sorghum, Zea, Allium, Phaseolus, Mangifera, Pachyrhizus* | - | Poisoning (Zinc phosphide, Aluminum phosphate, Thiodicarb) | - | |
| Mammals / Rodentia | *Oryzomys laticeps* | 1 | *Manihot, Theobroma* | - | - | Hunting (Undetermined, Traps), Acoustic deterrents (firecrackers, firearms) | |
| Mammals / Rodentia | *Oryzomys melanotis* | 1 | - | - | - | - | |
| Mammals / Rodentia | *Otospermophilus variegatus* | 1 | *Zea, Phaseolus, Avena* | - | Poisoning (Zinc phosphide, Aluminum phosphate, Thiodicarb), Hunting (Traps) | - | |
| Mammals / Rodentia | *Pappogeomys merriami* | 2 | *Zea, Avena, Phaseolus, Citrullus, Malus, Brassica, Solanum, Allium, Medicago* | - | Poisoning (Zinc phosphide, Aluminum phosphate, Thiodicarb), Hunting (Firearms) | - | |
| Mammals / Rodentia | *Peromyscus aztecus* | 1 | - | - | - | - | |
| Mammals / Rodentia | *Peromyscus boylii* | 1 | *Saccharum, Sorghum, Avena, Zea* | - | Poisoning (Zinc phosphide, Aluminum phosphate, Thiodicarb) | - | |
| Mammals / Rodentia | *Peromyscus difficilis* | 1 | *Zea, Saccharum, Phaseolus, Avena, Oryza* | - | Poisoning (Zinc phosphide, Aluminum phosphate, Thiodicarb) | - | |
| Mammals / Rodentia | *Peromyscus leucopus* | 2 | *Saccharum, Oryza* | - | Poisoning (Zinc phosphide, Aluminum phosphate, Thiodicarb) | - | |
| Mammals / Rodentia | *Peromyscus levipes* | 1 | - | - | - | - | |
| Mammals / Rodentia | *Peromyscus maniculatus* | 1 | - | - | - | - | |
| Mammals / Rodentia | *Peromyscus mexicanus* | 1 | *Zea* | - | - | - | |
| Mammals / Rodentia | *Phyllotis darwini* | 1 | *-* | - | - | Poisoning | |
| Mammals / Rodentia | *Rattus norvegicus* | 2 | *Zea, Saccharum*, *Theobroma, Citrullus, Sorghum* | - | Poisoning (Zinc phosphide, Aluminum phosphate, Thiodicarb) | Poisoning (Zinc phosphide, Diphacinone) | |
| Mammals / Rodentia | *Rattus rattus* | 6 | *Zea, Saccharum*, *Theobroma*, *Cocos, Triticum, Sorghum, Cucumis, Malus* | Poisoning (Pyriminil, Coumarin and Diphacinone), Agricultural practices (Field clearing) | Physical barriers (Metal bands), Poisoning (Zinc phosphide, Aluminum phosphate, Thiodicarb) | Poisoning (Undetermined, Zinc phosphide, Diphacinone) | |
| Mammals / Rodentia | *Reithrodontomys fulvescens* | 1 | - | - | - | - | |
| Mammals / Rodentia | *Reithrodontomys megalotis* | 2 | *Phaseolus, Zea, Saccharum* | - | Poisoning (Zinc phosphide, Aluminum phosphate, Thiodicarb) | - | |
| Mammals / Rodentia | *Reithrodontomys mexicanus* | 1 | - | - | - | - | |
| Mammals / Rodentia | *Reithrodontomys sumichrasti* | 1 | - | - | - | - | |
| Mammals / Rodentia | Sciuridae | 1 | *Zea*, *Phaseolus* | - | - | Poisoning (Herbicides) | |
| Mammals / Rodentia | *Sciurus aestuans* | 1 | *Theobroma* | - | - | Hunting (Undetermined, Traps), Acoustic deterrents (firecrackers, firearms) | |
| Mammals / Rodentia | *Sciurus aureogaster* | 3 | *Zea* | - | - | Hunting (weapons), Poisoning, Chemical repellents (Soap), Visual deterrents (Reflective objects, Scarecrows), Acoustic deterrents (Firecrackers), Vigilance (People, Guard dogs) | |
| Mammals / Rodentia | *Sciurus granatensis* | 2 | *Musa* ,*Theobroma*, *Cocos*, *Daucus*, *Zea*, *Oryza* | - | Palatable deterrents (Chile), Olfactory deterrents (Creolina) | Hunting (Firearms, Traps) | |
| Mammals / Rodentia | *Sciurus* sp. | 2 | *Manihot, Zea, Oryza, Sorghum, Saccharum* | Hunting, Agricultural practices (Field clearing), Olfactory deterrents (Human odors), Visual deterrents (Flags), Vigilance (People) | Poisoning (Zinc phosphide, Aluminum phosphate, Thiodicarb), Hunting (Traps) | - | |
| Mammals / Rodentia | *Sciurus variegatoides* | 2 | *Musa*, *Theobroma*, *Cocos*, *Daucus*, *Zea*, *Oryza*, *Carica*, *Persea*, *Mangifera*, *Pisum*, *Macadamia*, *Sechium* | - | - | Hunting (Firearms, Traps) | |
| Mammals / Rodentia | *Sigmodon alstoni* | 1 | *Oryza*, *Zea*, *Saccharum*, *Theobroma*, *Cucumis*, *Ipomoea*, *Ananas* | Poisoning (Biorat) | - | Poisoning (Zinc phosphide and Diphacinone) | |
| Mammals / Rodentia | *Sigmodon hirsutus* | 1 | *Arachis* | - | - | - | |
| Mammals / Rodentia | *Sigmodon hispidus* | 8 | *Ananas, Cucumis, Ipomoea*, *Coffea*, *Phaseolus*, *Arachis*, *Sorghum*, *Lycopersicon*, *Oryza*, *Saccharum*, *Zea*, *Elaeis, Solanum, Triticum, Cicer, Medicago, Cocos, Citrullus, Malus, Cucumis, Opuntia, Mangifera, Pachyrhizus* | Poisoning (Biorat) | Poisoning (Zinc phosphide, Aluminum phosphate, Thiodicarb) | Poisoning (Zinc phosphide, Thallium sulfate, Endrin, Coumatetralyl, Brodifacoum), Biological control (Attracting predators) | |
| Mammals / Rodentia | *Thomomys umbrinus* | 1 | *-* | - | - | - | |
| Mammals / Rodentia | *Zygodontomys brevicauda* | 3 | *Oryza*, *Elaeis*, *Zea*, *Shorgum* | - | - | - | |
| Birds / Accipitriformes | *Cathartes aura* | 1 | *Elaeis* | - | - | - | |
| Birds / Accipitriformes | *Coragyps atratus* | 1 | *Musa, Elaeis* | - | - | - | |
| Birds / Anseriformes | *Anas discors* | 1 | *Oryza* | - | - | - | |
| Birds / Anseriformes | *Cairina moschata* | 1 | *Zea, Oryza* | - | - | - | |
| Birds / Anseriformes | *Chloephaga picta* | 2 | *Triticum, Avena, Hordeum, Secale, Glycine, Helianthus, Zea, Sorghum* | - | - | Hunting | |
| Birds / Anseriformes | *Chloephaga poliocephala* | 2 | *Triticum, Avena, Hordeum, Secale, Glycine, Helianthus, Zea, Sorghum* | - | - | Hunting | |
| Birds / Anseriformes | *Chloephaga rubidiceps* | 2 | *Triticum, Avena, Hordeum, Secale, Glycine, Helianthus, Zea, Sorghum* | - | - | Hunting | |
| Birds / Anseriformes | *Chloephaga* sp. | 1 | *Triticum* | - | - | - | |
| Birds / Anseriformes | *Dendrocygna autumnalis* | 2 | *Oryza* | - | - | - | |
| Birds / Anseriformes | *Dendrocygna bicolor* | 1 | *Oryza* | - | - | - | |
| Birds / Anseriformes | *Dendrocygna* sp. | 1 | *Oryza* | - | - | - | |
| Birds / Anseriformes | *Dendrocygna viduata* | 1 | *Oryza* | - | - | - | |
| Birds / Anseriformes | *Netta* sp. | 1 | *Oryza* | - | - | - | |
| Birds / Cariamiformes | *Cariama cristata* | 1 | *Zea* | - | Acoustic deterrents (Firecrackers, Gas cannon), Visual deterrents (Scarecrows, Reflective objects), Vigilance (People), Physical barriers (Fencing, Netting), Chemical repellents, Hunting, Agricultural practices (Providing alternative food sources) | - | |
| Birds / Charadriiformes | *Jacana jacana* | 1 | *Oryza* | - | - | - | |
| Birds / Charadriiformes | *Larus maculipennis* | 1 | *-* | - | - | - | |
| Birds / Columbiformes | *Columba livia* | 1 | *Vitis* | Hunting (Firearms), Poisoning (Carbofuran), Visual deterrents (Flags, Scarecrows), Acoustic deterrents (Fireworks, Cannons, Distress calls), Chemical repellents (Methiocarb, Anthraquinone) | - | - | |
| Birds / Columbiformes | *Columbina inca* | 1 | *Oryza* | - | Poisoning (Organophosphates), Hunting (Firearms), Acustic deterrents (Fireworks, Canons), Visual deterrents (Scarecrows) | - | |
| Birds / Columbiformes | *Columbina minuta* | 2 | *Sorghum, Oryza* | - | - | - | |
| Birds / Columbiformes | *Columbina passerina* | 4 | *Sorghum, Zea, Oryza* | - | Poisoning (Organophosphates), Hunting (Firearms), Acustic deterrents (Fireworks, Canons), Visual deterrents (Scarecrows) | - | |
| Birds / Columbiformes | *Columbina squammata* | 1 | *Sorghum* | - | - | - | |
| Birds / Columbiformes | *Columbina talpacoti* | 3 | *Sorghum, Oryza* | - | - | - | |
| Birds / Columbiformes | *Leptotila verreauxi* | 1 | *Chenopodium* | Visual deterrents (Reflective objects), Acoustic deterrents, Chemical repellents (Bidrim) | - | - | |
| Birds / Columbiformes | *Metriopelia ceciliae* | 2 | *Chenopodium* | Visual deterrents (Reflective objects), Acoustic deterrents, Chemical repellents (Bidrim) | - | - | |
| Birds / Columbiformes | *Metriopelia melanoptera* | 1 | *Chenopodium* | - | - | - | |
| Birds / Columbiformes | *Patagioenas araucana* | 1 | *-* | - | - | - | |
| Birds / Columbiformes | *Patagioenas fasciata* | 1 | *Oryza, Pisum, Triticum* | - | - | - | |
| Birds / Columbiformes | *Patagioenas flavirostris* | 2 | *Sorghum, Citrus, Zea* | - | Poisoning (Organophosphates), Hunting (Firearms), Acustic deterrents (Fireworks, Canons), Visual deterrents (Scarecrows) | - | |
| Birds / Columbiformes | *Patagioenas maculosa* | 6 | *Chenopodium, Glycine*, *Helianthus*, *Triticum*, *Sorghum*, *Zea*, *Oryza*, *Hordeum* | - | - | Poisoning (Carbofuran, Parathion, Chlorpyrifos, Monocrotophos, Endrin, Mevinphos, Dicrotophos, CPT, CPTH), Hunting (Firearms), Chemical repellents (Anthraquinone, Methiocarb, Trimethacarb, Dimethyl, Methyl anthranilate, Synergized aluminum ammonium sulfate, Copper oxalate, Copper oxychloride, Condensed tannins, Avitrol), Reproductive control (Sterilants), Biological control (Suitable habitat reduction), Agricultural practices (Time of harvest, Alternative food sources, Kind of crops) | |
| Birds / Columbiformes | *Patagioenas picazuro* | 7 | *Glycine*, *Helianthus*, *Triticum*, *Sorghum*, *Zea*, *Oryza*, *Hordeum* | Hunting (Firearms), Poisoning (Carbofuran), Visual deterrents (Flags, Scarecrows), Acoustic deterrents (Fireworks, Cannons, Distress calls), Chemical repellents (Methiocarb, Anthraquinone) | - | Poisoning (Carbofuran, Parathion, Chlorpyrifos, Monocrotophos, Endrin, Mevinphos, Dicrotophos, CPT, CPTH), Hunting (Firearms), Chemical repellents (Anthraquinone, Methiocarb, Trimethacarb, Dimethyl, Methyl anthranilate, Synergized aluminum ammonium sulfate, Copper oxalate, Copper oxychloride, Condensed tannins, Avitrol), Reproductive control (Sterilants), Biological control (Suitable habitat reduction), Agricultural practices (Time of harvest, Location of crops, Kind of crops) | |
| Birds / Columbiformes | *Patagioenas* sp. | 1 | *Sorghum, Zea* | - | Acoustic deterrents (Firecrackers, Gas cannon), Visual deterrents (Scarecrows, Reflective objects), Vigilance (People), Physical barriers (Fencing, Netting), Chemical repellents, Hunting, Agricultural practices (Providing alternative food sources) | - | |
| Birds / Columbiformes | *Zenaida asiatica* | 3 | *Sorghum, Citrus, Triticum* | - | Poisoning (Organophosphates), Hunting (Firearms), Acustic deterrents (Fireworks, Canons), Visual deterrents (Scarecrows) | - | |
| Birds / Columbiformes | *Zenaida auriculata* | 15 | *Chenopodium, Glycine*, *Helianthus*, *Triticum*, *Sorghum*, *Zea*, *Oryza*, *Hordeum*, *Panicum*, *Avena*, *Brassica*,  *Vitis* | Visual deterrents (Reflective objects, Calcium carbonate paint, Flags, Scarecrows), Acoustic deterrents (Fireworks, Cannons, Distress calls, Chemical repellents (Bidrim, Methiocarb, Anthraquinone), Hunting (Firearms), Poisoning (Carbofuran), | Poisoning | Poisoning (Carbofuran, Parathion, Chlorpyrifos, Monocrotophos, Endrin, Mevinphos, Dicrotophos, CPT, CPTH), Hunting (Firearms), Chemical repellents (Anthraquinone, Methiocarb, Trimethacarb, Dimethyl, Methyl anthranilate, Synergized aluminum ammonium sulfate, Copper oxalate, Copper oxychloride, Condensed tannins, Avitrol), Reproductive control (Sterilants), Biological control (Suitable habitat reduction), Agricultural practices (Time of harvest, Kind of crops, Alternative food sources, Location of crops) | |
| Birds / Columbiformes | *Zenaida macroura* | 3 | *Oryza*, *Phaseolus*, *Zea*, *Sorghum* | - | Poisoning (Organophosphates), Hunting (Firearms), Acustic deterrents (Fireworks, Canons), Visual deterrents (Scarecrows) | - | |
| Birds / Cuculiformes | *Crotophaga ani* | 1 | *Sorghum* | - | - | Agricultural practices (Time of harvest, Location of crops) | |
| Birds / Galliformes | *Colinus virginianus* | 1 | *Zea* | - | Poisoning (Organophosphates), Hunting (Firearms), Acustic deterrents (Fireworks, Canons), Visual deterrents (Scarecrows) | - | |
| Birds / Galliformes | *Penelope obscura* | 2 | *Phaseolus, Zea*, *Vitis* | Hunting (Firearms), Poisoning (Carbofuran), Visual deterrents (Flags, Scarecrows), Acoustic deterrents (Fireworks, Cannons, Distress calls), Chemical repellents (Methiocarb, Anthraquinone) | Acoustic deterrents (Firecrackers, Gas cannon), Visual deterrents (Scarecrows, Reflective objects), Vigilance (People), Physical barriers (Fencing, Netting), Chemical repellents, Hunting, Agricultural practices (Providing alternative food sources) | - | |
| Birds / Galliformes | *Philortyx fasciatus* | 1 | *Zea* | - | Poisoning (Organophosphates), Hunting (Firearms), Acustic deterrents (Fireworks, Canons), Visual deterrents (Scarecrows) | - | |
| Birds / Gruiformes | *Aramides saracura* | 1 | *Zea* | - | Acoustic deterrents (Firecrackers, Gas cannon), Visual deterrents (Scarecrows, Reflective objects), Vigilance (People), Physical barriers (Fencing, Netting), Chemical repellents, Hunting, Agricultural practices (Providing alternative food sources) | - | |
| Birds / Gruiformes | *Gallinula galeata* | 1 | *Oryza, Zea* | - | - | - | |
| Birds / Gruiformes | *Grus canadensis* | 1 | *Zea*, *Avena*, *Sorghum*, *Triticum* | - | - | Acoustic deterrents, Visual deterrents (Scarecrows), Agricultural practices (Time of harvest), Hunting (Firearms) | |
| Birds / Gruiformes | *Porphyrio martinicus* | 1 | *Oryza* | - | - | - | |
| Birds / Passeriformes | *Agelaius phoeniceus* | 3 | *Zea, Sorghum, Oryza, Citrus* | - | Poisoning (Organophosphates), Hunting (Firearms), Acustic deterrents (Fireworks, Canons), Visual deterrents (Scarecrows) | - | |
| Birds / Passeriformes | *Carpodacus mexicanus* | 1 | *Sorghum* | - | Poisoning (Organophosphates), Hunting (Firearms), Acustic deterrents (Fireworks, Canons), Visual deterrents (Scarecrows) | - | |
| Birds / Passeriformes | *Chondestes grammacus* | 2 | *Sorghum* | - | Poisoning (Organophosphates), Hunting (Firearms), Acustic deterrents (Fireworks, Canons), Visual deterrents (Scarecrows) | - | |
| Birds / Passeriformes | *Chrysomus ruficapillus* | 2 | *Oryza* | - | - | Poisoning (Parathion) | |
| Birds / Passeriformes | *Corvus cryptoleucus* | 1 | *Sorghum, Citrus* | - | Poisoning (Organophosphates), Hunting (Firearms), Acustic deterrents (Fireworks, Canons), Visual deterrents (Scarecrows) | - | |
| Birds / Passeriformes | *Curaeus curaeus* | 1 | *-* | - | - | - | |
| Birds / Passeriformes | *Cyanocorax cristatellus* | 1 | *Zea* | - | Acoustic deterrents (Firecrackers, Gas cannon), Visual deterrents (Scarecrows, Reflective objects), Vigilance (People), Physical barriers (Fencing, Netting), Chemical repellents, Hunting, Agricultural practices (Providing alternative food sources) | Chemical repellents (Methiocarb), Agricultural practices (Field clearing) | |
| Birds / Passeriformes | *Cyanocorax yncas* | 1 | *Zea* | - | Palatable deterrents (Chile), Olfactory deterrents (Creolina) | - | |
| Birds / Passeriformes | *Diuca diuca* | 1 | *-* | - | - | - | |
| Birds / Passeriformes | *Dives atroviolaceus* | 1 | *Sorghum* | - | - | - | |
| Birds / Passeriformes | *Dives dives* | 2 | *Zea, Sorghum* | - | - | Hunting (weapons), Poisoning, Chemical repellents (Soap), Visual deterrents (Reflective objects, Scarecrows), Acoustic deterrents (Firecrackers), Vigilance (People, Guard dogs) | |
| Birds / Passeriformes | *Dolichonyx oryzivorus* | 3 | *Glycine, Oryza, Sorghum* | Acoustic deterrents (Firecrackers, Firearms, Yelling), Visual deterrents (Smoke) | - | Biological control (Attracting Predators), Poisoning, Visual deterrents (Reflective objects) | |
| Birds / Passeriformes | *Emberizoides herbicola* | 1 | *Sorghum* | - | - | - | |
| Birds / Passeriformes | *Furnarius rufus* | 1 | *Vitis* | Hunting (Firearms), Poisoning (Carbofuran), Visual deterrents (Flags, Scarecrows), Acoustic deterrents (Fireworks, Cannons, Distress calls), Chemical repellents (Methiocarb, Anthraquinone) | - | - | |
| Birds / Passeriformes | *Geospizopsis plebejus* | 1 | *Chenopodium* | - | - | - | |
| Birds / Passeriformes | *Gnorimopsar chopi* | 3 | *Oryza, Zea*, *Sorgum* | Vigilance (People, Guard dogs), Acoustic deterrents (Yelling, Firearms), Visual deterrents (Scarecrows, Fire), Agricultural practices (Early planting) | Acoustic deterrents (Firecrackers, Gas cannon), Visual deterrents (Scarecrows, Reflective objects), Vigilance (People), Physical barriers (Fencing, Netting), Chemical repellents, Hunting, Agricultural practices (Providing alternative food sources) | Agricultural practices (Time of harvest, Location of crops) | |
| Birds / Passeriformes | *Icterus chrysater* | 1 | *Zea* | - | Palatable deterrents (Chile), Olfactory deterrents (Creolina) | - | |
| Birds / Passeriformes | *Icterus galbula* | 1 | *Musa, Solaum, Citrus* | - | - | - | |
| Birds / Passeriformes | *Icterus pectoralis* | 1 | *Sorghum, Zea, Oryza* | - | Poisoning (Organophosphates), Hunting (Firearms), Acustic deterrents (Fireworks, Canons), Visual deterrents (Scarecrows) | - | |
| Birds / Passeriformes | *Leistes loyca* | 1 | *-* | - | - | - | |
| Birds / Passeriformes | *Leistes militaris* | 1 | *Sorghum, Oryza* | - | - | - | |
| Birds / Passeriformes | *Lonchura malacca* | 1 | *Sorghum* | - | - | - | |
| Birds / Passeriformes | *Lonchura punctulata* | 1 | *Sorghum* | - | - | - | |
| Birds / Passeriformes | *Mimus gilvus* | 1 | *Zea* | - | Palatable deterrents (Chile), Olfactory deterrents (Creolina) | - | |
| Birds / Passeriformes | *Mimus saturninus* | 2 | *Ficus*, *Vitis* | Hunting (Firearms), Poisoning (Carbofuran), Visual deterrents (Flags, Scarecrows), Acoustic deterrents (Fireworks, Cannons, Distress calls), Chemical repellents (Methiocarb, Anthraquinone) | - | - | |
| Birds / Passeriformes | *Molothrus aeneus* | 3 | *Zea, Solanum, Sorghum, Oryza, Citrus* | - | Poisoning (Organophosphates), Hunting (Firearms), Acustic deterrents (Fireworks, Canons), Visual deterrents (Scarecrows) | - | |
| Birds / Passeriformes | *Molothrus ater* | 2 | *Sorghum, Zea, Oryza* | - | Poisoning (Organophosphates), Hunting (Firearms), Acustic deterrents (Fireworks, Canons), Visual deterrents (Scarecrows) | - | |
| Birds / Passeriformes | *Molothrus bonariensis* | 2 | *Vitis* | Hunting (Firearms), Poisoning (Carbofuran), Visual deterrents (Flags, Scarecrows), Acoustic deterrents (Fireworks, Cannons, Distress calls), Chemical repellents (Methiocarb, Anthraquinone) | - | - | |
| Birds / Passeriformes | *Molothrus* sp. | 1 | *Glycine*, *Helianthus*, *Triticum*, *Sorghum*, *Oryza*, *Zea* | - | - | Poisoning (Parathion) | |
| Birds / Passeriformes | *Paroaria coronata* | 1 | *Ficus* | - | - | - | |
| Birds / Passeriformes | *Passer domesticus* | 6 | *Ficus*, *Sorghum*, *Vitis, Phaseolus, Zea, Manihot, Oryza, Tritichum* | Hunting (Firearms), Poisoning (Carbofuran), Visual deterrents (Flags, Scarecrows), Acoustic deterrents (Fireworks, Cannons, Distress calls), Chemical repellents (Methiocarb, Anthraquinone) | Poisoning (Organophosphates), Hunting (Firearms), Acustic deterrents (Fireworks, Canons), Visual deterrents (Scarecrows) | Hunting (Firearms), Poisoning, Biological control (Predators) | |
| Birds / Passeriformes | *Passerina caerulea* | 3 | *Sorghum, Triticum, Oryza, Fragaria, Zea* | - | Poisoning (Organophosphates), Hunting (Firearms), Acustic deterrents (Fireworks, Canons), Visual deterrents (Scarecrows) | - | |
| Birds / Passeriformes | *Passerina ciris* | 1 | *Sorghum* | - | - | - | |
| Birds / Passeriformes | *Passerina cyanea* | 2 | *Sorghum* | - | - | - | |
| Birds / Passeriformes | *Pheucticus aureoventris* | 1 | *Zea* | - | Palatable deterrents (Chile), Olfactory deterrents (Creolina) | - | |
| Birds / Passeriformes | *Pheucticus ludovicianus* | 1 | *Sorghum* | - | - | - | |
| Birds / Passeriformes | *Phrygilus punensis* | 1 | *Chenopodium* | - | - | - | |
| Birds / Passeriformes | *Phytotoma rara* | 1 | *-* | - | - | - | |
| Birds / Passeriformes | *Pipraeidea bonariensis* | 1 | *Ficus* | - | - | - | |
| Birds / Passeriformes | *Piranga flava* | 1 | *Citrus* | - | - | - | |
| Birds / Passeriformes | *Pitangus sulphuratus* | 3 | *Ficus*, *Vitis* | Hunting (Firearms), Poisoning (Carbofuran), Visual deterrents (Flags, Scarecrows), Acoustic deterrents (Fireworks, Cannons, Distress calls), Chemical repellents (Methiocarb, Anthraquinone) | - | - | |
| Birds / Passeriformes | *Psarocolius montezuma* | 2 | *Zea, Musa, Bactris* | - | - | Hunting (weapons), Poisoning, Chemical repellents (Soap), Visual deterrents (Reflective objects, Scarecrows), Acoustic deterrents (Firecrackers), Vigilance (People, Guard dogs) | |
| Birds / Passeriformes | *Pseudoleistes* sp. | 1 | *Glycine*, *Helianthus*, *Triticum*, *Sorghum*, *Oryza*, *Zea* | - | - | - | |
| Birds / Passeriformes | *Psilorhinus morio* | 3 | *Zea, Pisum, Persea, Cucurbita* | - | - | Hunting (weapons), Poisoning, Chemical repellents (Soap), Visual deterrents (Reflective objects, Scarecrows), Acoustic deterrents (Firecrackers), Vigilance (People, Guard dogs) | |
| Birds / Passeriformes | *Quiscalus mexicanus* | 4 | *Zea, Sorghum, Triticum, Oryza, Fragaria, Citrus, Cucumis* | - | Poisoning (Organophosphates), Hunting (Firearms), Acustic deterrents (Fireworks, Canons), Visual deterrents (Scarecrows) | Hunting (weapons), Poisoning, Chemical repellents (Soap), Visual deterrents (Reflective objects, Scarecrows), Acoustic deterrents (Firecrackers), Vigilance (People, Guard dogs) | |
| Birds / Passeriformes | *Rhopospina fruticeti* | 1 | *Chenopodium* | - | - | - | |
| Birds / Passeriformes | *Saltator coerulescens* | 2 | *Ficus, Sorghum* | - | - | - | |
| Birds / Passeriformes | *Sicalis flaveola* | 1 | *Sorghum* | - | - | Agricultural practices (Time of harvest, Location of crops) | |
| Birds / Passeriformes | *Sicalis luteola* | 2 | *Chenopodium, Sorghum* | - | - | - | |
| Birds / Passeriformes | *Sicalis* sp. | 1 | *Glycine*, *Helianthus*, *Triticum*, *Sorghum*, *Oryza*, *Zea* | - | - | - | |
| Birds / Passeriformes | *Sicalis uropigyalis* | 1 | *Chenopodium* | - | - | - | |
| Birds / Passeriformes | *Spinus atratus* | 1 | *Chenopodium* | - | - | - | |
| Birds / Passeriformes | *Spinus psaltria* | 1 | *Sorghum* | - | Poisoning (Organophosphates), Hunting (Firearms), Acustic deterrents (Fireworks, Canons), Visual deterrents (Scarecrows) | - | |
| Birds / Passeriformes | *Spinus spinescens* | 1 | *Chenopodium* | Visual deterrents (Reflective objects), Acoustic deterrents, Chemical repellents (Bidrim) | - | - | |
| Birds / Passeriformes | *Spiza americana* | 4 | *Oryza, Sorghum* | Chemical repellent (Anthraquinone, Methiocarb), Poisoning, Acoustic deterrents (Firecrackers, Sirens, Horns, Yelling, Firearms), Visual deterrents (Flags, Scarecrows, Reflective objects, Smoke), Biological control (Attracting predators) | Chemical repellent (Methyl anthranilate) | Poisoning, Hunting (Firearms) | |
| Birds / Passeriformes | *Sporophila lineola* | 1 | *Sorghum* | - | - | Agricultural practices (Time of harvest, Location of crops) | |
| Birds / Passeriformes | *Sporophila minuta* | 1 | *Sorghum, Oryza* | - | - | - | |
| Birds / Passeriformes | *Sporophila nigricollis* | 1 | *Sorghum* | - | - | Agricultural practices (Time of harvest, Location of crops) | |
| Birds / Passeriformes | *Sporophila* sp. | 1 | *Sorghum* | - | - | Agricultural practices (Time of harvest, Location of crops) | |
| Birds / Passeriformes | *Sporophila torqueola* | 2 | *Sorghum, Oryza* | - | Poisoning (Organophosphates), Hunting (Firearms), Acustic deterrents (Fireworks, Canons), Visual deterrents (Scarecrows) | - | |
| Birds / Passeriformes | *Sturnus vulgaris* | 1 | *Vaccinium*, *Morus*, *Prunus* | - | - | - | |
| Birds / Passeriformes | *Thamnophilus doliatus* | 1 | *Elettaria* | - | - | - | |
| Birds / Passeriformes | *Thraupis episcopus* | 2 | *Zea, Malus* | - | Palatable deterrents (Chile), Olfactory deterrents (Creolina) | - | |
| Birds / Passeriformes | *Thraupis sayaca* | 1 | *Ficus* | - | - | - | |
| Birds / Passeriformes | *Thraupis* sp. | 1 | *-* | - | Acoustic deterrents (Firecrackers, Gas cannon), Visual deterrents (Scarecrows, Reflective objects), Vigilance (People), Physical barriers (Fencing, Netting), Chemical repellents, Hunting, Agricultural practices (Providing alternative food sources) | - | |
| Birds / Passeriformes | *Toxostoma curvirostre* | 1 | *Sorghum, Triticum, Oryza, Fragaria* | - | Poisoning (Organophosphates), Hunting (Firearms), Acustic deterrents (Fireworks, Canons), Visual deterrents (Scarecrows) | - | |
| Birds / Passeriformes | *Turdus amaurochalinus* | 2 | *Ficus*, *Vitis* | Hunting (Firearms), Poisoning (Carbofuran), Visual deterrents (Flags, Scarecrows), Acoustic deterrents (Fireworks, Cannons, Distress calls), Chemical repellents (Methiocarb, Anthraquinone) | - | - | |
| Birds / Passeriformes | *Turdus chiguanco* | 2 | *Chenopodium*, *Zea* | - | - | Hunting, Acoustic deterrents (Fireworks), Vigilance (People) | |
| Birds / Passeriformes | *Turdus falcklandii* | 1 | *-* | - | - | - | |
| Birds / Passeriformes | *Turdus fuscater* | 1 | *Zea* | - | Palatable deterrents (Chile), Olfactory deterrents (Creolina) | - | |
| Birds / Passeriformes | *Turdus rufiventris* | 2 | *Vitis* | Hunting (Firearms), Poisoning (Carbofuran), Visual deterrents (Flags, Scarecrows), Acoustic deterrents (Fireworks, Cannons, Distress calls), Chemical repellents (Methiocarb, Anthraquinone) | - | - | |
| Birds / Passeriformes | *Tyrannus melancholicus* | 1 | *Sorghum* | - | - | Agricultural practices (Time of harvest, Location of crops) | |
| Birds / Passeriformes | *Tyrannus savana* | 1 | *Vitis* | Hunting (Firearms), Poisoning (Carbofuran), Visual deterrents (Flags, Scarecrows), Acoustic deterrents (Fireworks, Cannons, Distress calls), Chemical repellents (Methiocarb, Anthraquinone) | - | - | |
| Birds / Passeriformes | *Volatinia jacarina* | 3 | *Oryza*, *Sorghum* | - | - | Agricultural practices (Time of harvest, Location of crops) | |
| Birds / Passeriformes | *Xanthocephalus xanthocephalus* | 2 | *Sorghum, Oryza, Triticum, Cicer, Phaseolus* | - | Poisoning (Organophosphates), Hunting (Firearms), Acustic deterrents (Fireworks, Canons), Visual deterrents (Scarecrows) | - | |
| Birds / Passeriformes | *Zonotrichia capensis* | 4 | *Chenopodium*, *Vitis* | Visual deterrents (Reflective objects, Flags, Scarecrows), Hunting (Firearms), Poisoning (Carbofuran), Acoustic deterrents (Fireworks, Cannons, Distress calls), Chemical repellents (Methiocarb, Anthraquinone, Bidrim) | - | - | |
| Birds / Passeriformes | *Zonotrichia leucophrys* | 1 | *Sorghum* | - | - | - | |
| Birds / Pelecaniformes | *Bubulcus ibis* | 1 | *Oryza* | - | - | - | |
| Birds / Piciformes | *Colaptes campestris* | 1 | *Vitis* | Hunting (Firearms), Poisoning (Carbofuran), Visual deterrents (Flags, Scarecrows), Acoustic deterrents (Fireworks, Cannons, Distress calls), Chemical repellents (Methiocarb, Anthraquinone) | - | - | |
| Birds / Piciformes | *Hylatomus lineatus* | 2 | *Zea* | - | - | - | |
| Birds / Piciformes | *Melanerpes candidus* | 1 | *Zea* | Vigilance (People, Guard dogs), Acoustic deterrents (Yelling, Firearms), Visual deterrents (Scarecrows, Fire), Agricultural practices (Early planting) | - | - | |
| Birds / Piciformes | *Melanerpes chrysauchen* | 1 | *Musa* | - | - | - | |
| Birds / Piciformes | *Melanerpes formicivorus* | 1 | *Zea* | - | - | - | |
| Birds / Piciformes | *Melanerpes striatus* | 1 | *Theobroma* | - | Hunting, Chemical repellents (Methiocarb), Visual deterrents (Carpenter’s chalk), Olfactory deterrents (Tabebuia extract) | - | |
| Birds / Piciformes | *Ramphastos toco* | 1 | *-* | - | Acoustic deterrents (Firecrackers, Gas cannon), Visual deterrents (Scarecrows, Reflective objects), Vigilance (People), Physical barriers (Fencing, Netting), Chemical repellents, Hunting, Agricultural practices (Providing alternative food sources) | - | |
| Birds / Psittaciformes | *Amazona aestiva* | 1 | *Citrus* | - | - | - | |
| Birds / Psittaciformes | *Amazona albifrons* | 2 | *Zea, Mangifera* | - | - | Hunting (weapons), Poisoning, Chemical repellents (Soap), Visual deterrents (Reflective objects, Scarecrows), Acoustic deterrents (Firecrackers), Vigilance (People, Guard dogs) | |
| Birds / Psittaciformes | *Amazona autumnalis* | 1 | *Sorghum, Mangifera, Citrus, Persea, Helianthus* | - | - | - | |
| Birds / Psittaciformes | *Amazona* sp. | 1 | *Oryza* | - | Poisoning (Organophosphates), Hunting (Firearms), Acustic deterrents (Fireworks, Canons), Visual deterrents (Scarecrows) | - | |
| Birds / Psittaciformes | *Ara* sp. | 1 | *Bertholletia* | - | - | - | |
| Birds / Psittaciformes | *Aratinga* sp. | 1 | *Zea* | - | - | Hunting, Acoustic deterrents (Fireworks), Vigilance (People) | |
| Birds / Psittaciformes | *Brotogeris chiriri* | 2 | *Zea*, *Sorghum* | Vigilance (People, Guard dogs), Acoustic deterrents (Yelling, Firearms), Visual deterrents (Scarecrows, Fire), Agricultural practices (Early planting) | - | Agricultural practices (Time of harvest, Location of crops) | |
| Birds / Psittaciformes | *Brotogeris jugularis* | 1 | *Zea* | - | - | - | |
| Birds / Psittaciformes | *Cyanoliseus patagonus* | 1 | *Avena, Helianthus, Triticum, Zea* | - | - | - | |
| Birds / Psittaciformes | *Diopsittaca nobilis* | 1 | *Sorghum* | - | - | Agricultural practices (Time of harvest, Location of crops) | |
| Birds / Psittaciformes | *Eupsittula aurea* | 1 | *Zea* | Vigilance (People, Guard dogs), Acoustic deterrents (Yelling, Firearms), Visual deterrents (Scarecrows, Fire), Agricultural practices (Early planting) | - | - | |
| Birds / Psittaciformes | *Eupsittula canicularis* | 1 | *Zea, Sorghum* | - | - | - | |
| Birds / Psittaciformes | *Eupsittula nana* | 1 | *Zea* | - | Poisoning (Organophosphates), Hunting (Firearms), Acustic deterrents (Fireworks, Canons), Visual deterrents (Scarecrows) | - | |
| Birds / Psittaciformes | *Eupsittula pertinax* | 1 | *Zea, Sorghum, Sesamum* | - | - | - | |
| Birds / Psittaciformes | *Forpus xanthopterygius* | 1 | *Sorghum* | - | - | Agricultural practices (Time of harvest, Location of crops) | |
| Birds / Psittaciformes | *Myiopsitta monachus* | 11 | *Glycine, Helianthus, Medicago, Panicum, Sorghum, Triticum, Zea*, *Oryza*, *Ficus*, *Citrus*, *Prunus*, *Vaccinium*, *Vitis* | Reproductive control (Nest destruction), Hunting (Firearms), Poisoning (Carbofuran), Visual deterrents (Flags, Scarecrows), Acoustic deterrents (Fireworks, Cannons, Distress calls), Chemical repellents (Methiocarb, Anthraquinone) | Chemical repellents, Physical barriers, Agricultural practices (Early planting, Field clearing, Providing alternative food sources), Capture and relocation | Poisoning (Carbofuran, Parathion, Chlorpyrifos, Monocrotophos, Endrin, Mevinphos, Dicrotophos, CPT, CPTH, Insecticides), Hunting (Firearms, Traps), Chemical repellents (Methiocarb, Trimethacarb, Dimethyl, Methyl anthranilate, Synergized aluminum ammonium sulfate, Copper oxalate, Copper oxychloride, Condensed tannins, Avitrol), Reproductive control (Nest burning, Egg destruction, Sterilants), Agricultural practices (Kind of crops, Time of harvest, Location of crops, Field clearing, Crop density, Alternative food sources), Biological control (Suitable habitat reduction), Acoustic deterrents (Cannons, Fireworks, Predator sounds), Visual deterrents (Reflective objects, Predator outlines, Balloons), Vigilance (People), Capture and relocation | |
| Birds / Psittaciformes | *Pionus maximiliani* | 1 | *Zea* | - | - | - | |
| Birds / Psittaciformes | *Pionus menstruus* | 1 | *Zea, Musa, Bactris* | - | - | - | |
| Birds / Psittaciformes | *Pionus senilis* | 2 | *Zea, Bactris, Sorghum, Oryza* | - | - | - | |
| Birds / Psittaciformes | *Psittacara finschi* | 1 | *Zea, Sorghum* | - | - | - | |
| Birds / Psittaciformes | *Psittacara leucophthalmus* | 2 | *Sorghum, Zea* | - | Acoustic deterrents (Firecrackers, Gas cannon), Visual deterrents (Scarecrows, Reflective objects), Vigilance (People), Physical barriers (Fencing, Netting), Chemical repellents, Hunting, Agricultural practices (Providing alternative food sources) | Agricultural practices (Time of harvest, Location of crops) | |
| Birds / Psittaciformes | Psittacidae | 2 | *Zea* | - | - | Vigilance | |
| Birds / Strigiformes | *Athene cunicularia* | 1 | *Sorghum* | - | - | Agricultural practices (Time of harvest, Location of crops) | |
| Reptiles / Squamata | *Iguana iguana* | 1 | *Dioscorea*, *Xanthosoma*, *Cucurbita*, *Cucumis* | - | - | - | |
