# Supplementary material for: Crop damage by vertebrates in Latin America: current knowledge and potential future management directions
Source: PeerJ. 2022 Mar 25;10:e13185. doi: 10.7717/peerj.13185 (PMC8958972; doi:10.7717/peerj.13185)
Supplement: Supplemental Information 3 — Including information on the crop taxa in each study, the vertebrate taxa that interact with them, the protection techniques used, and the efficiency of the protection techniques. [file peerj-10-13185-s003.docx]

**Crop damage by vertebrates in Latin America: current knowledge and potential future management directions**

**Adrián Alejandro Cuesta Hermira, Fernanda Michalski**

Supplementary Material

**Table S3: List of the 113 reviewed studies with species and crop protection data.**

Including information on the crop taxa in each study, the vertebrate taxa that interact with them, the protection techniques used, and the efficiency of the protection techniques.

| **Study** | **Crop taxa** | **Vertebrate taxa** | **Protection techniques** | **Efficiency** |
| --- | --- | --- | --- | --- |
| Abba et al. (2015) | *Glycine max*, *Zea mays*, *Helianthus annuus*, *Triticum aestivum* | *Chaetophractus villosus*, *Dasypus hybridus* | None | - |
| Abrahams et al. (2018) | *Manihot esculenta* | *Dasyprocta fuliginosa*, *Pecari tajacu*, *Cuniculus paca*, *Mazama americana*, Echimyidae | Hunting (weapons, dogs, traps), Vigilance (People), Visual deterrents (Scarecrows), Agricultural practices (Field clearing, Firebreaks), Physical barriers (Netting), Acoustic deterrents (Yelling) | Not quantified |
| Aguiar et al. (2011) | *Citrullus* sp., *Persea americana*, *Morus nigra*, *Hovenia dulcis*, *Eriobotrya japonica*, *Vassobia breviflora*, *Musa* sp. | *Nasua nasua*, *Procyon cancrivorus* | None | - |
| Albarracín and Aliaga-Rossel (2018) | *Zea mays* | *Tremarctos ornatus*, *Aratinga* sp., *Turdus chiguanco*, *Conepatus chinga* | Hunting, Acoustic deterrents (Fireworks), Vigilance (People) | Not quantified |
| de Almeida-Jácomo et al. (2013) | *Zea* sp., *Glycine* sp., *Sorghum* sp., *Panicum* sp. | *Tayassu pecari* | Hunting | Not quantified |
| Aris et al. (2008) | Undetermined | *Conepatus chinga* | Hunting | Not quantified |
| Arroyo-Quiroz et al. (2017) | *Zea* sp., *Phaseolus* sp., *Arachis* sp., *Carica* sp., *Persea* sp., *Mangifera* sp., *Musa* sp., *Cucurbita* sp., *Cicer* sp., *Pisum* sp. | Sciuridae, Leporidae, Muridae, Psittacidae, *Nasua narica*, *Urocyon cinereoargenteus*, *Odocoileus virginianus*, *Procyon lotor*, *Dasypus novemcinctus*, *Didelphis virginiana*, *Rattus rattus* | Poisoning (Herbicides) | Not quantified |
| Avery et al. (2001) | *Oryza sativa* | *Spiza americana* | Chemical repellent (Anthraquinone, Methyl anthranilate, Methiocarb) | Varying |
| Barceló et al. (2012) | *Zea* sp., *Avena* sp., *Sorgum* sp., *Triticum* sp. | *Grus canadensis* | Acoustic deterrents, Visual deterrents (Scarecrows), Agricultural practices (Time of harvest), Hunting (Firearms) | Not quantified |
| Basili and Temple (1999a) | *Oryza* sp., *Sorghum* sp. | *Spiza americana* | Poisoning, Acoustic deterrents (Firecrackers, Sirens, Horns, Yelling, Firearms), Visual deterrents (Flags, Scarecrows, Reflective objects, Smoke), Biological control (Attracting predators) | Effective |
| Basili and Temple (1999b) | *Oryza* sp., *Sorghum* sp. | *Spiza americana* | Poisoning, Hunting (Firearms) | Not quantified |
| Berón et al. (2020) | *Ficus carica* | *Myiopsitta monachus*, *Pitangus sulphuratus*, *Mimus saturninus*, *Turdus amaurochalinus*, *Turdus rufiventri*, *Thraupis sayaca*, *Pipraeidea bonariensis*, *Paroaria coronate*, *Saltator coerulescens*, *Passer domesticus* | None | - |
| Bou et al. (2016) | *Glycine max* | *Zenaida auriculata*, *Patagioenas picazuro*, *Patagioenas maculosa* | None | - |
| Boulton et al. (1996) | *Annona* sp., *Mangifera* sp., *Carica* sp., *Psidium* sp., *Arachis* sp., *Malus* sp., *Pisum* sp., *Musa* sp., *Prunus* sp., *Zea* sp., *Cucumis* sp., *Blighia* sp., *Manihot* sp., *Perse asp*., *Daucus* sp., *Ipomoea* sp., *Cucurbita* sp., *Solanum* sp., *Artocarpus* sp., *Phaseolus* sp., *Saccharum* sp., *Abelmoschus* sp., *Dioscorea* sp., *Citrus* sp., *Brassica* sp., *Allium* sp., *Beta* sp. | *Chlorocebus aethiops* | Hunting (Firearms, Traps) | Not effective |
| Bourne (1981) | *Oryza sativa* | *Dendrocygna autumnalis* | None | - |
| Bruggers et al. (1998) | *Glycine* sp., *Helianthus* sp., *Triticum* sp., *Sorghum* sp., *Zea* sp., *Oryza* sp., *Citrus* sp., *Hordeum* sp., *Malus* sp., *Pyrus* sp., *Prunus* sp. | *Zenaida auriculata*, *Patagioenas picazuro*, *Patagioenas maculosa*, *Myiopsitta monachus*, *Molothrus* sp., *Chrysomus ruficapillus*, *Pseudoleistes* sp., *Sicalis* sp., *Chloephaga* sp., *Dendrocygna* sp., *Netta* sp., *Amazona aestiva* | Poisoning (Carbofuran, Parathion, Chlorpyrifos, Monocrotophos, Endrin, Mevinphos, Dicrotophos, CPT, CPTH), Hunting (Firearms), Chemical repellents (Methiocarb, Trimethacarb, Dimethyl, Methyl anthranilate, Synergized aluminum ammonium sulfate, Copper oxalate, Copper oxychloride, Condensed tannins, Avitrol), Reproductive control (Sterilants), Agricultural practices (Kind of crops, Time of harvest), Biological control (Suitable habitat reduction) | Not quantified |
| Bucher and Aramburú (2014) | *Sorghum* sp., *Helianthus* sp., *Zea* sp. | *Myiopsitta monachus* | None | - |
| Bucher and Ranvaud (2006) | *Sorghum* sp., *Helianthus* sp., *Oryza* sp., *Zea* sp., *Tritichum* sp., *Hordeum* sp., *Glycine* sp. | *Zenaida auriculata* | Poisoning | Not effective |
| Calamari et al. (2018) | Undetermined | *Zenaida auriculata*, *Myiopsitta monachus* | None | - |
| Canavelli et al. (2012) | *Zea* sp., *Helianthus* sp., *Sorgum* sp., *Triticum* sp., *Oryza* sp., *Citrus* sp., *Prunus* sp., *Vaccinium* sp. | *Myiopsitta monachus* | Hunting (Firearms, Traps), Poisoning (Insecticides), Reproductive control (Nest burning, Egg destruction, Sterilants), Agricultural practices (Time of harvest, Location of crops, Kind of crops, Field clearing), Acoustic repellents (Canons, Fireworks, Predator sounds) | Not quantified |
| Canavelli et al. (2013) | *Zea* sp., *Helianthus* sp., *Glycine* sp., *Triticum* sp., *Sorghum* sp., *Medicago* sp., *Panicum* sp. | *Myiopsitta monachus* | Hunting (Weapons, Traps), Poisoning, Reproductive control (Nest destruction), Chemical repellents, Physical barriers, Agricultural practices (Early planting, Field clearing, Providing alternative food sources), Capture and relocation | Varying |
| Canavelli et al. (2014) | *Zea* sp., *Helianthus* sp. | *Myiopsitta monachus* | Agricultural practices (Time of harvest, Crop density, Kind of crops) | Not quantified |
| Can-Hernandez et al. (2019) | *Zea mays* | *Quiscalus mexicanus*, *Psilorhinus morio*, *Psarocolius montezuma*, *Amazona albifrons*, *Dives dives*, *Nasua narica*, *Procyon lotor*, Procyonidae, *Sciurus aureogaster*, *Cuniculus paca*, *Odocoileus virginianus* | Hunting (Weapons), Poisoning, Chemical repellent (Soap), Visual deterrents (Reflective objects, Scarecrows), Acoustic deterrents (Firecrackers), Vigilance (People, Guard dogs) | Not quantified |
| de Carvalho et al. (2019) | *Zea mays*, *Phaseolus* sp., *Sorghum bicolor*, *Oryza sativa*, *Saccharum* sp., Fruits, Vegetables | *Psittacara leucophthalmus*, *Hydrochoerus hydrochaeris*, *Penelope obscura*, *Patagioenas* spp., *Sus scrofa*, *Nasua nasua*, *Thraupis* spp., *Ramphastos toco*, *Aramides saracura*, *Gnorimopsar chopi*, *Cyanocorax cristatellus*, *Cariama cristata*, *Dasypodidae* spp., *Didelphis* sp., *Allouatta* sp. | Acoustic deterrents (Firecrackers, Gas cannon), Visual deterrents (Scarecrows, Reflective objects), Vigilance, Physical barriers (Netting, Fencing), Chemical repellents, Hunting, Agricultural practices (Providing alternative food sources) | Not effective |
| Castilho et al. (2018) | *Zea* sp., *Manihot* sp. | *Pecari tajacu*, *Hydrochoerus hydrochaeris* | Hunting | Not quantified |
| Castillo-Chinchilla et al. (2018) | Undetermined | *Procyon lotor*, *Nasua narica*, *Odocoileus virginianus*, *Cebus capucinus*, *Alouatta palliata*, *Mustela frenata*, *Sylvilagus floridanus*, *Sciurus variegatoides* | None | - |
| Castillo-Lopez et al. (2017) | *Zea mays* | *Sciurus granatensis*, *Chilomys instans*, *Didelphis marsupialis*, *Cyanocorax yncas*, *Icterus chrysater*, *Turdus fuscater*, *Mimus gilvus*, *Pheucticus aureoventris*, *Thraupis episcopus*, Leporidae, Chiroptera | Palatable deterrent (Chile), Olfactory deterrent (Creolina) | Not effective |
| Cervo and Guadagnin (2020) | *Avena sativa*, *Sorghum bicolor*, *Lolium* sp., *Zea mays*, *Oryza sativa*, *Glycine max* | *Sus scrofa* | Hunting (Weapons, Dogs, Traps) | Not quantified |
| Chaves and Bicca-Marques (2017) | *Psidium guajava*, *Eriobotrya japonica*, *Diospyros kaki*, *Citrus reticulata*, *Araucaria angustifolia*, *Citrus sinensis* | *Alouatta guariba clamitans* | None | - |
| Cirne and López-Iborra (2005) | *Oryza sativa* | *Agelaius ruficapillus* | None | - |
| Codesido et al. (2015) | *Glycine max*, *Zea mays*, *Helianthus annus* | *Zenaida auriculata*, *Myiopsitta monachus*, *Patagioenas picazuro*, *Patagioenas maculosa* | None | - |
| Cornejo (2000) | *Saccharum* sp. | *Orthogeomys hispidus* | Poisoning (Sodium monofuroacetate) | Effective |
| Corrêa et al. (2018) | *Morus nigra*, *Eriobotrya japonica*, *Psidium guajava*, *Syzygium cumini*, *Hovenia dulcis*, *Melia azedarach*, *Ligustrum lucidum* | *Alouatta guariba* | None | - |
| Cossios et al. (2018) | Undetermined | *Conepatus chinga* | Hunting | Not quantified |
| Costán & Sarasola (2017) | *Panicum miliaceum*, *Triticum aestivum*, *Helianthus annus*, *Avena sativa*, *Zea may*, *Sorghum bicolor* | *Zenaida auriculata* | None | - |
| Dardanelli et al. (2016) | *Glycine max*, *Triticum* sp., *Zea* sp., *Brassica* sp. | *Zenaida auriculata*, *Patagioenas maculosa*, *Patagioenas picazuro*, *Myiopsitta monachus* | Agricultural practices (Harvest time, Alternative food sources) | Not quantified |
| Dore et al. (2018) | Undetermined | *Chlorocebus aethiops* | Vigilance (Dogs) | Not quantified |
| Doutel-Ribas et al. (2019) | Undetermined | *Sus scrofa* | Vigilance (Guard dogs) | Not effective |
| Eiris and Barreto (2009) | *Oryza* sp. | *Holochilus sciureus* | Poisoning (Rodenticides, Organophosphates) | Not quantified |
| Engeman et al. (2010) | *Cucurbita* sp., *Citrullus* sp., *Cucumis* sp., *Carica* sp., *Musa* sp., *Zea* sp. | *Macaca mulatta*, *Erythrocebus patas* | Agricultural practices (Kind of crops) | Not quantified |
| Escobar-Lasso et al. (2020) | *Musa sapientum*, *Musa paradisiaca* | *Tremarctos ornatus* | Fencing | Not effective |
| Felix et al. (2014) | *Oryza sativa*, *Zea mays*, *Saccharum*, *Glycine max* | *Hydrochoerus hydrochaeris* | None | - |
| Ferraz et al. (2003) | *Zea mays* | *Hydrochoerus hydrochaeris* | None | - |
| Ferraz et al. (2007) | *Saccharum* sp. | *Hydrochoerus hydrochaeris* | None | - |
| Ferraz et al. (2009) | *Saccharum* sp. | *Hydrochoerus hydrochaeris* | None | - |
| Figueroa (2013) | *Annona cherimola*, *Ananas comosus*, *Opuntia ficus-indica*, *Cucurbita pepo*, *Cucurbita maxima*, *Cucurbita moschata*, *Manihot esculenta*, *Persea americana*, *Psidium guajava*, *Musa paradisiaca*, *Musa sapientum*, *Saccharum officinarum*, *Zea mays*, *Citrus* sp., *Lucuma obovata*, *Solanum quitoense*, *Solanum tuberosum* | *Tremarctos ornatus* | Hunting | Not quantified |
| Flores-Armillas et al. (2020) | *Zea mays* | *Nasua narica*, *Odocoileus virginianus*, Birds | None | - |
| de Freitas et al. (2008) | *Zea mays*, *Saccharum officinarum* | *Sapajus libidinosus* | None | - |
| Fuentes and Campusano (1985) | Undetermined | *Abrothrix olivacea*, *Mus musculus*, *Octodon degus*, *Oligoryzomys longicaudatus*, *Phyllotis darwini*, *Rattus rattus*, *Lepus europaeus* | Poisoning | Not quantified |
| Galetti (1993) | *Zea mays* | *Pionus maximiliani* | None - |  |
| García and Peiró (2016) | *Oryza sativa*, *Phaseolus vulgaris*, *Zea mays* | *Zenaida macroura* | None | - |
| García-Mendoza and Prieto-Rosales (2019) | *Zea mays* | Psittacidae | Vigilance | Not quantified |
| Gonzalez and Acosta-Perez (2002) | *Oryza* sp. | *Molothrus aeneus*, *Molothrus ater*, *Quiscalus mexicanus*, *Agelaius phoeniceus*, *Passerina* *caerulea*, *Volatinia jacarina* | None | - |
| Gorosábel et al. (2019) | *Triticum* sp. | *Chloephaga rubidiceps*, *Chloephaga poliocephala*, *Chloephaga picta* | None | - |
| Hilje (1992) | *Persea americana*, *Oryza sativa*, *Pisum sativum*, *Avena sativa*, *Musa paradisiaca*, *Theobroma cacao*, *Coffea arabica*, *Saccharum officinarum*, *Allium cepa*, *Cocos nucifera*, *Sechium edule*, *Phaseolus* *vulgaris*, *Macadamia* *integriflora*, *Zea* *mays*, *Colocasia* *esculenta*, *Mangifera* *indica*, *Arachis* *hypogaea*, *Elaeis* *guineensis*, *Solanum* *tuberosum*, *Carica* *papaya*, *Bactris* *gasipaes*, *Ananas* *comosus*, *Musa* *paradisiaca*, *Brassica* *oleracea*, *Sorghum* *bicolor*, *Tamarindus* *indica*, *Xanthosoma* *violaceum*, *Manihot* *esculenta*, *Lycopersicon* *esculentum*, *Daucus* *carota*, *Cucurbita* *moschata* | *Orthogeomys cavator*, *Orthogeomys* *cherriei*, *Orthogeomys* *heterodus*, *Orthogeomys* *underwoodi*, *Sigmodon* *hispidus*, *Sciurus* *granatensis*, *Sciurus* *variegatoides* | Poisoning (Metomil, Zinc phosphide, Thallium sulfate, Endrin, Coumatetralyl, Brodifacoum), Hunting (Firearms, Traps), Agricultural practices (Field clearing), Biological control (Attracting predators) | Not quantified |
| Horrocks and Baulu (1988) | Undetermined | *Chlorocebus aethiops* | Hunting (Traps) | Not effective |
| Horrocks and Baulu (1994) | *Annona* sp., *Mangifera* sp., *Spondias* sp., *Carica* sp., *Psidium* sp., *Arachis* sp., *Passiflora* sp., *Malus* sp., *Pisum* sp., *Musa* sp., *Prunus* sp., *Zea* sp., *Cucumis* sp., *Blighia* sp., *Manihot* sp., *Persea* sp., *Daucus* sp., *Ipomoea* sp., *Cucurbita* sp., *Solanum* sp., *Artocarpus* sp., *Phaseolus* sp., *Abelmoschus* sp., *Dioscorea* sp., *Citrus* sp., *Colocasia* sp., *Brassica* sp., *Allium* sp., *Beta* sp., *Saccharum* sp. | *Chlorocebus aethiops* | Hunting (Traps), Agricultural practices (Kind of crops, Location of crops, Alternative food sources, Field clearing), | Not quantified |
| Horváth et al. (2001) | *Zea* sp., *Phaseolus* sp., *Solanum* sp., *Coffea* sp. | *Sigmodon hispidus* | None | - |
| Ibañez et al. (2016) | *Vaccinium* sp., *Morus* sp., *Prunus* sp. | *Sturnus vulgaris* | None | - |
| Jackson (1988) | *Saccharum* sp., *Oryza* sp., *Zea* sp., *Panicum* sp., *Sorghum* sp., *Helianthus* sp., *Citrus* sp. | *Lepus europaeus*, *Holochilus brasiliensis*, *Calomys musculinus*, *Calomys laucha*, *Oligoryzomys nigripes*, *Akodon azarae*, *Ctenomys* sp., *Cavia aperea*, *Galea musteloides*, *Chaetophractus* sp., *Dasypus* sp., *Sus scrofa*, *Tayassu pecari*, *Pecari tajacu* | None | - |
| Key and de la Piedra Constantino (1992) | *Zea* sp., *Saccharum* sp., *Theobroma* sp., *Coffea* sp. | *Rattus rattus*, *Rattus* *norvegicus*, *Sigmodon* *hispidus*, *Orthogeomys* sp. | Poisoning (Zinc phosphide and Diphacinone) | Not quantified |
| Lima et al. (2019) | *Zea mays*, *Glycine max* | *Tayassu pecari* | Hunting (Weapons, Dogs, Traps), Poisoning (Carbofuran), Physical barriers (Electric fencing, Trenches), Agricultural practices (Providing alternative food sources, Barrier crops), Acoustic deterrents (Firecrackers) | Varying |
| Lins and Ferreira (2018) | *Saccharum* sp. | *Sapajus flavius* | None | - |
| Lobão and Nogueira-Filho (2011) | *Theobroma cacao*, *Manihot esculenta*, *Musa* sp., *Phaseolus* sp., *Zea mays*, *Bactris gasipaes*, *Carica papaya*, *Elaeis* sp. | *Oryzomys laticeps*, *Pecari tajacu*, *Cuniculus paca*, *Metachirus nudicaudatus*, *Nectomys squamipes*, *Sciurus aestuans*, *Dasyprocta aguti*, *Hydrochoerus hydrochaeris*, *Nasua nasua*, *Callistomys pictus*, *Leontopithecus chrysomelas*, *Procyon cancrivorus*, *Didelphis aurita*, *Euphractus sexcinctus*, *Dasypus novemcinctus*, *Cabassous unicinctus* | Hunting (Weapons, Traps), Acoustic deterrents (Firecrackers, Firearms) | Not quantified |
| López-Torres et al. (2012) | *Dioscorea* sp., *Xanthosoma* sp., *Cucurbita* sp., *Cucumis* sp. | *Iguana iguana* | None | - |
| Loza-del-Carpio et al. (2016) | *Chenopodium quinoa* | *Patagioenas maculosa*, *Sicalis uropigyalis*, *Zenaida auriculata*, *Zonotrichia capensis*, *Geospizopsis plebejus*, *Phrygilus punensis*, *Rhopospina fruticeti*, *Sicalis luteola*, *Metriopelia melanoptera*, *Turdus chiguanco*, *Metriopelia ceciliae*, *Spinus atratus* | None | - |
| MacGregor-Fors et al. (2011) | Undetermined | *Myiopsitta monachus* | Hunting (Firearms, Traps), Reproductive control (Sterilants) | Not quantified |
| Marchand (2016) | Undetermined | *Pecari tajacu*, *Dasyprocta* spp., *Hydrochoerus hydrochaeris*, *Mazama* spp. | Agricultural practices (Field clearing), Physical barriers (Fencing) | Not effective |
| McKinney (2011) | *Elaeis guineensis*, *Cocos nucifera*, *Musa acuminata* | *Cebus capucinus* | None | - |
| McKinney (2019) | *Mangifera indica* | *Alouatta palliata* | None | - |
| Melo and Cheschini (2012) | *Sorghum bicolor* | *Athene cunicularia*, *Patagioenas picazuro*, *Columbina* *talpacoti*, *Zenaida* *auriculata*, *Crotophaga* *ani*, *Diopsittaca* *nobilis*, *Psittacara* *leucophthalmus*, *Brotogeris* *chiriri*, *Forpus* *xanthopterygius*, *Tyrannus* *melancholicus*, *Sporophila* *lineola*, *Sporophila* *nigricollis*, *Sporophila* sp., *Volatinia* *jacarina*, *Sicalis* *flaveola*, *Gnorimopsar* *chopi* | Agricultural practices (Time of harvest, Location of crops) | Not quantified |
| Mendonça et al. (2011) | *Zea* sp. | *Cerdocyon thous* | Hunting | Not quantified |
| Mitchell and Bruggers (1985) | *Theobroma cacao* | *Melanerpes striatus* | Hunting, Chemical repellents (Methiocarb), Visual deterrents (Carpenter’s chalk), Olfactory deterrents (Tabebuia extract) | Not effective/  Inconclusive |
| Monge (1999) | *Bactris gasipaes* | *Orthogeomys cherriei*, *Orthogeomys heterodus*, *Orthogeomys cavator*, *Orthogeomys underwoodi* | None | - |
| Monge (2013) | *Oryza* sp., *Zea* sp., *Musa*, *Elaeis* sp., *Triticum* sp., *Sorghum* sp., *Sesamum* sp., *Bactris* sp., *Mangifera* sp., *Citrus* sp., *Persea* sp., *Helianthus* sp., *Elettaria* sp., *Vitis* sp., *Malus* sp., *Cucurbita* sp., *Pisum* sp., *Cicer* sp., *Phaseolus* sp., *Cucumis* sp., *Solanum* sp. | *Dendrocygna* viduata, Dendrocygna *autumnalis*, *Dendrocygna bicolor*, *Cairina* moschata, Anas *discors* , *Bubulcus ibis*, *Coragyps atratus*, *Cathartes aura*, *Porphyrio martinicus*, *Gallinula galeata*, *Jacana jacana*, *Patagioenas flavirostris*, *Patagioenas fasciata*, *Zenaida asiatica*, *Columbina passerina*, *Columbina minuta*, *Columbina talpacoti*, *Psittacara finschi*, *Eupsittula canicularis*, *Eupsittula pertinax*, *Brotogeris jugularis*, *Pionus menstruus*, *Pionus senilis*, *Amazona albifrons*, *Amazona autumnalis*, *Melanerpes formicivorus*, *Melanerpes chrysauchen*, *Dryocopus lineatus*, *Thamnophilus doliatus*, *Pitangus sulphuratus*, *Psilorhinus morio*, *Thraupis episcopus*, *Saltator coerulescens*, *Volatinia jacarina*, *Sporophila torqueola*, *Sporophila minuta*, *Sicalis luteola*, *Emberizoides herbicola*, *Chondestes grammacus*, *Zonotrichia capensis*, *Piranga flava*, *Pheucticus ludovicianus*, *Passerina caerulea*, *Passerina cyanea*, *Passerina ciris*, *Spiza americana*, *Dolichonyx oryzivorus*, *Agelaius phoeniceus*, *Leistes militaris*, *Xanthocephalus xanthocephalus*, *Dives dives*, *Quiscalus mexicanus*, *Molothrus aeneus*, *Icterus galbula*, *Psarocolius montezuma*, *Passer domesticus* | None | - |
| Monge-Meza (2011) | *Musa* sp. | *Orthogeomys cherriei*, *Orthogeomys heterodus*, *Orthogeomys cavator*, *Orthogeomys underwoodi* | Biological control (Infectious disease, Introduction of predators), Poisoning (Estricnina, Methyl bromide, Metomil, Aluminium phosphate), Hunting (Traps) | Varying |
| Monge-Meza and Orozco (2010) | *Ananas comusus* | *Philander opossum* | None | - |
| Monge-Meza et al. (2014) | *Arachis hypogaea* | *Sigmodon hirsutus* | None | - |
| Naughton-Treves et al. (2003) | Undetermined | *Tapirus terrestris*, *Eira barbara*, *Hydrochoerus hydrochaeris*, *Cuniculus paca*, *Pecari tajacu*, *Dasyprocta variegata* | Hunting | Not quantified |
| Olivera et al. (2016) | *Glycine max* | *Zenaida auriculata*, *Patagioenas maculosa*, *Patagioenas picazuro* | Chemical repellents (Anthraquinone, Methiocarb, Methyl anthranilate) | Not quantified |
| Parra et al. (2012) | *Oryza sativa* | *Holochilus sciureus*, *Zygodontomys brevicauda*, *Sigmodon alstoni*, *Oligoryzomys* sp. | None | - |
| Pedrana et al. (2014) | *Triticum aestivum*, *Avena sativa*, *Hordeum vulgare*, *Secale cereale*, *Glycine max*, *Helianthus annuus*, *Zea mays*, *Sorghum graniferum* | *Chloephaga rubidiceps*, *Chloephaga poliocephala*, *Chloephaga picta* | Hunting | Not quantified |
| Pedrosa et al. (2015) | *Zea mays* | *Sus scrofa* | Hunting | Not quantified |
| Pereira et al. (2019) | *Zea mays*, *Saccharum* sp., *Daucus carota*, *Fragaria* sp., *Cucurbita* sp. | *Sus scrofa* | Hunting | Not quantified |
| Pérez and Bulla (2000) | *Sorghum vulgare* | *Columbina minuta*, *Columbina passerina*, *Columbina talpacoti*, *Columbina squammata* | None | - |
| Pérez and Pacheco (2006) | *Manihot esculenta*, *Colocasia esculenta*, *Xanthosoma* sp., *Zea mays* | *Dasyprocta variegata*, *Pecari tajacu*, *Cuniculus paca*, *Nasua nasua*, *Sapajus apella*, Birds, Muridae | Physical barriers (Wire mesh exclosures) | Effective |
| Pérez and Pacheco (2014) | *Manihot esculenta*, *Xanthosoma* sp., *Zea mays* | *Pecari tajacu*, *Dasyprocta punctata*, *Cuniculus paca*, *Dinomys branickii*, *Nasua nasua*, *Didelphis* sp., *Sciurus* sp., *Sapajus apella*, Rodentia, Birds | Hunting, Agricultural practices (Field clearing), Olfactory deterrents (Human odors), Visual deterrents (Flags), Vigilance (People) | Effective |
| Peyton (1980) | *Zea mays*, *Saccharum officinarum*, *Annona cherimolia*, *Cucurbita moschata*, *Lucuma obovata* | *Tremarctos ornatus* | Vigilance, Agricultural practices (Field clearing), Visual deterrents (Fire), Olfactory deterrents (Burnt rubber), Hunting | Not quantified |
| Poleo et al. (2010) | *Oryza sativa* | *Zygodontomys brevicauda* | None | - |
| Ranvaud et al. (2001) | *Zea mays*, *Oryza* *sativa*, *Triticum* *aestivum*, *Glycine* *max* | *Zenaida auriculata* | None | - |
| Renfrew and Saavedra (2007) | *Oryza sativa*, *Sorghum bicolor*, *Glycine max* | *Dolichonyx oryzivorus* | Acoustic deterrents (Firecrackers, Firearms, Yelling), Visual deterrents (Reflective objects, Smoke), Biological control (Attracting predators), Poisoning | Effective / Not quantified |
| Renfrew et al. (2017) | *Oryza sativa* | *Dolichonyx oryzivorus* | Poisoning | Not quantified |
| Robles et al. (2003) | *Chenopodium quinoa* | *Zenaida auriculata*, *Metriopelia* *ceciliae*, *Leptotila* *verreauxi*, *Spinus* *spinescens*, *Zonotrichia* *capensis* | Visual deterrents (Reflective objects), Acoustic deterrents, Chemical repellents (Bidrim) | Effective |
| Rocha and Fortes (2015) | *Zea mays* | *Sapajus nigritus* | Agricultural practices (Early planting, Crop location), Vigilance (Guard dogs), Acoustic deterrents | Varying |
| Rodriguez and Avery (1996) | *Oryza sativa* | *Chrysomus ruficapillus* | Chemical repellents (Methiocarb), Agricultural practices (Field clearing) | Not quantified |
| Rodriguez et al. (1995) | *Helianthus* sp. | *Zenaida auriculata* | Chemical repellents (Methiocarb), Visual deterrent (Calcium carbonate paint) | Effective |
| Rodriguez et al. (2004) | *Vitis* sp. | *Patagioenas picazuro*, Pitangus sulphuratus, *Turdus* *amaurochalinus*, *Passer* *domesticus*, *Mimus* *saturninus*, *Turdus* *rufiventris*, *Colaptes* *campestris*, *Zenaida* *auriculata*, *Columba* *livia*, *Zonotrichia* *capensis*, *Myiopsitta* *monachus*, *Furnarius* *rufus*, *Penelope* *obscura*, *Tyrannus* *savana*, *Molothrus* *bonariensis* | Hunting (Firearms), Poisoning (Carbofuran), Visual deterrents (Flags, Scarecrows), Acoustic deterrents (Fireworks, Cannons, Distress calls), Chemical repellents (Methiocarb, Anthraquinone) | Effective |
| Romero-Balderas et al. (2006) | *Zea mays* | *Procyon lotor*, *Pecari tajacu*, *Nasua narica*, *Cuniculus paca*, *Sciurus aureogaster*, *Orthogeomys hispidus*, *Peromyscus mexicanus*, *Pionus senilis*, *Dryocopus lineatus*, *Psilorhinus morio* | None | - |
| Rosa et al. (2018) | *Saccharum* sp., *Zea mays*, *Manihot esculenta* | *Sus scrofa* | Hunting (Weapons, Dogs, Traps) | Not quantified |
| Sanchez et al. (2016) | *Helianthus* sp., *Zea mays*, *Triticum* sp., *Avena sativa* | *Cyanoliseus patagonus* | None | - |
| Sanchez-Cordero and Martinez-Meyer (2000) | *Zea mays*, *Saccharum* sp., *Coffea* sp., *Phaseolus* sp., *Oryza sativa*, *Avena sativa*, *Sorghum bicolor*, *Tritichum* sp. | *Sciurus aureogaster*, *Microtus mexicanus*, *Oligoryzomys fulvescens*, *Oryzomys couesi*, *Oryzomys melanotis*, *Peromyscus aztecus*, *Peromyscus leucopus*, *Peromyscus levipes*, *Peromyscus maniculatus*, *Reithrodontomys fulvescens*, *Reithrodontomys megalotis*, *Reithrodontomys mexicanus*, *Reithrodontomys sumichrasti*, *Sigmodon hispidus*, *Orthogeomys hispidus*, *Pappogeomys merriami*, *Thomomys umbrinus* | None | - |
| Santos (2018) | *Oryza sativa* | *Holochilus sciureus* | Hunting (Dogs) | Not quantified |
| Saucedo et al. (2010) | *Sorghum bicolor* | *Passer domesticus*, *Lonchura* *malacca*, *Lonchura* *punctulata*, *Dives* *atroviolaceus*, *Passerina* *cyanea*, *Zonotrichia* *leucophrys*, *Columbina* *passerina*, *Zenaida* *macroura*, *Zenaida* *asiatica* | None | - |
| Silva-Andrade et al. (2016) | *Phaseolus vulgaris*, *Zea mays*, *Manihot esculenta*, Fruits | *Passer domesticus* | Hunting (Firearms), Poisoning, Biological control (Predators) | Not quantified |
| Silva-Rodríguez et al. (2006) | Undetermined | *Curaeus curaeus*, *Diuca diuca*, *Larus maculipennis*, *Molothrus bonariensis*, *Patagioenas araucana*, *Phytotoma rara*, *Leistes loyca*, *Turdus falcklandii*, *Zenaida auriculata* | None | - |
| Spagnoletti et al. (2017) | *Zea mays*, *Oryza* sp., *Phaseolus sp.*, *Manihot esculenta*, *Musa* sp., *Mangifera indica*, *Citrullus lanatu*, *Ananas comosus*, *Carica papaya* | *Sapajus libidinosus*, *Brotogeris chiriri*, *Gnorimopsar chopi*, *Melanerpes candidus*, *Eupsittula aurea* | Vigilance (People, Guard dogs), Acoustic deterrents (Yelling, Firearms), Visual deterrents (Scarecrows, Fire), Agricultural practices (Early planting) | Effective |
| Trivedi et al. (2004) | *Bertholletia excelsa* | *Ara* sp. | None | - |
| Valencia (1980) | *Cocos nucifera* | *Rattus rattus* | Poisoning (Pyriminil, Coumarin and Diphacinone), Agricultural practices (Field clearing), Physical barriers (Metal bands) | Varying |
| Valencia et al. (1994) | *Oryza* sp., *Cocos* sp., *Elaeis* sp., *Zea* sp., *Sorghum* sp. | *Rattus rattus*, *Holochilus* *brasiliensis*, *Sigmodon* *hispidus*, *Zygodontomys* *brevicauda* | None | - |
| Villa et al. (1998) | *Saccharum* sp. | *Sigmodon hispidus*, *Oryzomys* *couesi*, *Handleyomys* *chapmani* | None | - |
| Villafana-Martin et al. (1999) | *Cucumis sativus*, *Ipomoea batata*, *Ananas camusus* | *Sigmodon hispidus* | Poisoning (Biorat) | Effective |
| del Villar-González (2000) | *Zea* sp., *Phaseolus* sp., *Saccharum* sp., *Triticum* sp., *Medicago* sp., *Cicer* sp., *Cocos* sp., *Capsicum* sp., *Citrullus* sp., *Cucumis* sp., *Malus* sp., *Cucurbita* sp., *Brassica* sp., *Avena* sp., *Opuntia* sp., *Sorghum* sp., *Oryza* sp., *Pachyrhizus* sp., *Mangifera* sp., *Solanum* sp., *Allium* sp., *Carya* sp., *Citrus* sp., *Fragaria* sp. | *Sigmodon hispidus*, *Mus musculus*, *Peromyscus leucopus*, *Rattus norvegicus*, *Rattus rattus*, *Peromyscus difficilis*, *Peromyscus boylii*, *Liomys irroratus*, *Reithrodontomys megalotis*, *Oryzomys couesi*, *Microtus mexicanus*, *Pappogeomys merriami*, *Cratogeomys fumosus*, *Notocitellus adocetus*, *Otospermophilus variegatus*, *Sciurus* sp., *Lepus* sp., *Corvus cryptoleucus*, *Molothrus aeneus*, *Agelaius phoeniceus*, *Quiscalus mexicanus*, *Patagioenas flavirostriss*, *Zenaida asiatica*, *Toxostoma curvirostre*, *Passerina caerulea*, *Sporophila torqueola*, *Chondestes grammacus*, *Carpodacus mexicanus*, *Spinus psaltria*, *Xanthocephalus xanthocephalus*, *Molothrus ater*, *Icterus pectoralis*, *Passer domesticus*, *Colinus virginianus*, *Philortyx fasciatus*, *Zenaida macroura*, *Columbina inca*, *Columbina passerina*, *Eupsittula nana*, *Amazona* sp. | Poisoning (Zinc phosphide, Aluminum phosphate, Thiodicarb, Organophosphates), Hunting (Traps, Firearms), Acustic deterrents (Fireworks, Canons), Visual deterrents (Scarecrows) | Not effective |
| Waters (2015) | *Phaseolus* sp., *Zea* *mays*, *Musa* sp., *Brassica oleracea*, *Ananas comosus*, *Solanum tuberosum*, *Citrullus lanatus*, *Dioscorea alata* | *Tapirus bairdii*, *Nasua narica*, *Procyon lotor*, *Tayassu* sp. | Hunting | Not quantified |

**References:**

Abba AM, Zufiaurre E, Codesido M, Bilenca DN. 2015. Burrowing activity by armadillos in agroecosystems of central Argentina: biogeography, land use, and rainfall effects. *Agriculture*, *Ecosystems & Environment* 200:54–61. DOI: 10.1016/j.agee.2014.11.001.

Abrahams MI, Peres CA, Costa HC. 2018. Manioc losses by terrestrial vertebrates in western Brazilian Amazonia. *The Journal of Wildlife Management* 82:734–746. DOI: 10.1002/jwmg.21443.

Aguiar LM, Moro-Rios RF, Silvestre T, Silva-Pereira JE, Bilski DR, Passos FC, Sekiama ML, Rocha VJ. 2011. Diet of brown-nosed coatis and crab-eating raccoons from a mosaic landscape with exotic plantations in southern Brazil. *Studies on Neotropical Fauna and Environment* 46:153–161. DOI: 10.1080/01650521.2011.640567.

Albarracín V, Aliaga-Rossel E. 2018. Bearly guilty: Understanding human–Andean bear conflict regarding crop losses. *Ethnobiology Letters* 9:323–332. DOI: 10.14237/ebl.9.2.2018.1300.

de Almeida Jácomo AT, Furtado MM, Kashivakura CK, Marinho-Filho J, Sollmann R, Tôrres NM, Silveira L. 2013. White-lipped peccary home-range size in a protected area and farmland in the central Brazilian grasslands. *Journal of Mammalogy* 94:137–145. DOI: 10.1644/11-MAMM-A-411.1.

Aris ID, León De Castro MW, Ruesta PV. 2008. Relaciones entre los pobladores rurales y los carnívoros altoandinos del distrito de Anco, centro-Sur del Perú. *Ecología Aplicada* 7:43–48. DOI: 10.21704/rea.v7i1-2.358.

Arroyo-Quiroz I, García-Barrios R, Argueta-Villamar A, Smith R, Pérez-Gil R. 2017. Local perspectives on conflicts with wildlife and their management in the Sierra Gorda Biosphere Reserve, Mexico. *Journal of Ethnobiology*, 37: 719-742. DOI: 10.2993/0278-0771-37.4.719

Avery ML, Tillman EA, Laukert CC. 2001. Evaluation of chemical repellents for reducing crop damage by Dickcissels in Venezuela. *International Journal of Pest Management* 47:311–314. DOI: 10.1080/09670870110065235.

Barceló I, Guzmán-Aranda JC, Chávez-Ramírez F, Powell LA. 2012. Rural Inhabitant Perceptions of Sandhill Cranes in Wintering Areas of Northern Mexico. *Human Dimensions of Wildlife* 17:301–307. DOI: 10.1080/10871209.2012.668610.

Basili GD, Temple SA. 1999a. Dickcissels and crop damage in Venezuela: Defining the problem with ecological models. *Ecological applications* 9:732–739. DOI: 10.1890/1051-0761(1999)009[0732:DACDIV]2.0.CO;2.

Basili GD, Temple SA. 1999b. Winter ecology, behavior, and conservation needs of Dickcissels in Venezuela. *Studies in Avian Biology*. 19:289–299.

Berón IJ, Bortoluzzi A, Dardanelli S. 2020. Avifauna de cuatro plantaciones de higo (*Ficus carica*) en el centro-este de Argentina. *Ornitología neotropical* 31:34–41.

Bou N, Dardanelli S, Olivera L, Tellechea G, Orduna LA, Canavelli S, Rodriguez E. 2016. Desarrollo de un método para evaluar el daño ocasionado por aves en cultivos comerciales de soja recién emergida. *Idesia* 34:67-74. DOI: 10.4067/S0718-34292016005000036.

Boulton AM, Horrocks JA, Baulu J. 1996. The Barbados vervet monkey (*Cercopithecus aethiops sabaeus*): Changes in population size and crop damage, 1980–1994. *International Journal of Primatology* 17:831-844. DOI: 10.1007/BF02735267.

Bourne GR. 1981. Food habits of Black-bellied Whistling Ducks occupying rice culture habitats. *The Wilson Bulletin* 93:551–554.

Bruggers RL, Rodriguez E, Zaccagnini, ME. 1998. Planning for bird pest problem resolution: A case study. *International Biodeterioration & Biodegradation* 42:173–184. DOI: 10.1016/S0964-8305(98)00046-8.

Bucher EH, Aramburú RM. 2014. Land-use changes and monk parakeet expansion in the Pampas grasslands of Argentina. *Journal of Biogeography* 41:1160–1170. DOI: 10.1111/jbi.12282.

Bucher EH, Ranvaud RD. 2006. Eared dove outbreaks in South America: patterns and characteristics. *Acta Zoologica Sinica* 52:564–567.

Calamari NC, Canavelli SB, Cerezo A, Dardanelli S, Bernardos JN, Zaccagnini ME. 2018. Variations in pest bird density in Argentinean agroecosystems in relation to land use and/or cover, vegetation productivity and climate. *Wildlife Research* 45:668–678. DOI: 10.1071/WR17167.

Canavelli SB, Aramburú R, Zaccagnini ME. 2012. Aspectos a considerar para disminuir los conflictos originados por los daños de la cotorra (*Myiopsitta monachus*) en cultivos agrícolas. *Hornero* 27:89-101.

Canavelli SB, Swisher ME, Branch LC. 2013. Factors related to farmers' preferences to decrease monk parakeet damage to crops. *Human Dimensions of Wildlife* 18:124-137. DOI: 10.1080/10871209.2013.745102.

Canavelli SB, Branch LC, Cavallero P, González C, Zaccagnini ME. 2014. Multi-level analysis of bird abundance and damage to crop fields. *Agriculture*, *Ecosystems and Environment* 197:128–136. DOI: 10.1016/j.agee.2014.07.024.

Can-Hernández G, Villanueva-García C, Gordillo-Chávez EJ, Pacheco-Figueroa CJ, Pérez-Netzahual E, García-Morales R. 2019. Wildlife damage to crops adjacent to a protected area in southeastern Mexico: farmers’ perceptions versus actual impact. *Human-Wildlife Interactions* 13:423–438. DOI: 10.26077/9gqj-5m75.

de Carvalho ALC, Araújo AR, Machado TMM, Ribon R, Lopes LE. 2019. Wildlife and damage to agriculture: an ethnobiological approach with rural producers in southeastern Brazil. *Revista Brasileira de Ornitologia* 27:17–26. DOI: 10.1007/bf03544442.

Castilho LC, de Vleeschouwer KM, Milner-Gulland EJ, Schiavetti A. 2018. Attitudes and behaviors of rural residents toward different motivations for hunting and deforestation in protected areas of the northeastern Atlantic forest, Brazil. *Tropical Conservation Science* 11:1–14. DOI: 10.1177/1940082917753507.

Castillo-Chinchilla M, Piedra-Castro L, Sandoval-Hernández I, Carvajal-Sánchez JP. 2018. Conocimiento popular de los mamíferos del Parque Nacional Barra Honda, Nicoya, Costa Rica. *Uniciencia* 32:82–95. DOI: 10.15359/ru.32-2.6.

Castillo-López IF, Rodríguez-Africano PE, Montes-Pérez RC, González-Valderrama DM. 2017. Fauna silvestre que afecta los cultivos en Boyacá y control del daño a cultivos de maíz. *Ciencia y Agricultura* 14:75–84. DOI: 10.19053/01228420.v14.n1.2017.6090.

Cervo IB, Guadagnin DL. 2020. Wild boar diet and its implications on agriculture and biodiversity in Brazilian forest–grassland ecoregions. *Animal Biodiversity and Conservation* 43:123–136. DOI: 10.32800/abc.2020.43.0123.

Chaves ÓM, Bicca-Marques JC. 2017. Crop feeding by brown howlers (*Alouatta guariba clamitans*) in forest fragments: the conservation value of cultivated species. *International Journal of Primatology* 38:263–281. DOI: 10.1007/s10764-016-9927-8.

Cirne MP, López-Iborra GM. 2005. Breeding biology of Chestnut-capped Blackbirds in rice paddies in southern Brazil. *Journal of Field Ornithology* 76:411–416. DOI: 10.1648/0273-8570-76.4.411.

Codesido M, Zufiaurre E, Bilenca D. 2015. Relationship between pest birds and landscape elements in the Pampas of Central Argentina. *Emu* 115:80–84. DOI: 10.1071/MU13110.

Cornejo BV. 2000. Pocket gopher (*Orthogeomys hispidus hispidus*) damage in sugarcane fields in the state of Veracruz, Mexico. *Proceedings of the Vertebrate Pest Conference* 19:358–361. DOI: 10.5070/V419110222

Corrêa FM, Chaves ÓM, Printes RC, Romanowski HP. 2018. Surviving in the urban–rural interface: Feeding and ranging behavior of brown howlers (*Alouatta guariba clamitans*) in an urban fragment in southern Brazil. *American Journal of Primatology* 80: e22865. DOI: 10.1002/ajp.22865.

Cossios ED, Ridoutt FV, Donoso AL. 2018. Relationships between Molina’s hog nosed skunks, *Conepatus chinga* (Mammalia, Mephitidae) and human beings in the Chaupihuaranga river basin, Pasco, Peru. *Ecología Aplicada* 17:207–214. DOI: 10.21704/rea.v17i2.1240.

Costán AS, Sarasola JH. 2017. Eared dove (*Zenaida auriculata*) granivory and its role in seed dispersal in semiarid forests of central Argentina. *Ornitología Neotropical* 28:43-50.

Dardanelli S, Fandiño B, Calamari NC, Canavelli SB, Zaccagnini ME. 2016. ¿Eligen las palomas y cotorras los lotes de soja (*Glycine max*) en emergencia? Un caso de estudio en agroecosistemas de Entre Ríos, Argentina. *Revista Mexicana de Biodiversidad* 87:1308–1314. DOI: 10.1016/j.rmb.2016.09.006.

Dore KM, Eller AR, Eller JL. 2018. Identity construction and symbolic association in farmer-vervet monkey (*Chlorocebus aethiops sabaeus*) interconnections in St. Kitts. *Folia Primatologica* 89:63–80. DOI: 10.1159/000479064.

Doutel-Ribas C, Martins FI, Campos Z, Piovezan U, Tomas W, Silva VS, Pellegrin A, Mourão G. 2019. Invasive wild boars and native mammals in agroecosystems in the Atlantic Forest of Western Brazil. *Pesquisa Agropecuária Brasileira* 54:e00241. DOI: 10.1590/S1678-3921.PAB2019.V54.00241.

Eiris GC, Barreto GR. 2009. Home range of mars rats, *Holochilus sciureus*, a rodent pest in rice fields of Venezuela. *Interciencia* 34:400–405.

Engeman RM, Laborde JE, Constantin BU, Shwiff SA, Hall P, Duffiney A, Luciano F. 2010. The economic impacts to commercial farms from invasive monkeys in Puerto Rico. *Crop Protection* 29:401–405. DOI: 10.1016/j.cropro.2009.10.021.

Escobar-Lasso S, Cepeda-Duque JC, Gil-Fernández M, González-Maya JF. 2020. Is the banana ripe? Andean bear-human conflict in a protected area of Colombia. *Human-Wildlife Interactions* 14:200–215. DOI: 10.26077/6e5e-089e.

Felix GA, Almeida Paz ICL, Piovezan U, Garcia RG, Lima KAO, Nääs IA, Salgado DD, Pilecco M, Belloni M. 2014. Feeding behavior and crop damage caused by capybaras (*Hydrochoerus hydrochaeris*) in an agricultural landscape. *Brazilian Journal of Biology* 74:779–786. DOI: 10.1590/1519-6984.02113.

Ferraz KMPMB, Lechevalier M-A, Couto HTZ, Verdade LM. 2003. Damage caused by capybaras in a corn field. *Scientia Agricola* 60:191–194. DOI: h10.1590/S0103-90162003000100029

Ferraz KMPM de B, Ferraz SF de B, Moreira JR, Couto HTZ, Verdade LM. 2007. Capybara (*Hydrochoerus* *hydrochaeris*) distribution in agroecosystems: a cross-scale habitat analysis. *Journal of Biogeography* 34:223–230. DOI: 10.1111/j.1365-2699.2006.01568.x.

Ferraz KMPMB, Peterson AT, Scachetti-Pereira R, Vettorazzi CA, Verdade LM. 2009. Distribution of capybaras in an agroecosystem, southeastern Brazil, based on ecological niche modeling. *Journal of Mammalogy* 90:189–194. DOI: 10.1644/07-MAMM-A-338.1.

Figueroa J. 2013. Revisión de la dieta del oso andino *Tremarctos ornatus* (Carnivora: Ursidae) en América del Sur y nuevos registros para el Perú. *Revista del Museo Argentino de Ciencias Naturales* 15: 1–27

Flores-Armillas VH, López-Medellín X, Barrios RG, MacGregor-Fors I, Valenzuela-Galván D. 2020. Landscape features associated with damage to Maize (*Zea mays*) fields in Central México: A comparison of wind and wildlife damage. *Agriculture* 10:460. DOI: 10.3390/agriculture10100460.

de Freitas CH, Setz EZF, Araújo ARB, Gobbi N. 2008. Agricultural crops in the diet of bearded capuchin monkeys, *Cebus libidinosus* Spix (Primates: Cebidae), in forest fragments in southeast Brazil. *Revista Brasileira de Zoologia* 25:32–39. DOI: 10.1590/S0101-81752008000100006.

Fuentes ER, Campusano C. 1985. Pest outbreaks and rainfall in the semi-arid region of Chile. *Journal of Arid Environments* 8:67–72. DOI: 10.1016/s0140-1963(18)31338-7.

Galetti M. 1993. Diet of the scaly-headed parrot (*Pionus maximiliani*) in a semideciduous forest in southeastern Brazil. *Biotropica* 25:419–425. DOI: 10.2307/2388865.

García JM, Peiró V. 2016. Feeding use of a tropical agroecosystem (Cuba) by Mourning dove (*Zenaida macroura* L). *The Journal of Animal and Plant Sciences* 26:1879–1885.

García-Mendoza PJ, Prieto-Rosales GP. 2019. Análisis preliminar de los daños ocasionados al maíz por vertebrados plagas en la localidad Pilcos, Colcabamba, Perú. *Tayacaja* 2:111–126. DOI: 10.46908/rict.v2i1.43.

González DV, Acosta Perez N. 2002. Assessment of bird damage to early-ripening rice in Cuautla, Morelos State, Mexico. *Proceedings of the Vertebrate Pest Conference* 20:157–160. DOI: 10.5070/v420110237.

Gorosábel A, Pedrana J, Bernad L, Caballero VJ, Muñoz SD, Maceira NO. 2019. Evaluating the impacts and benefits of sheldgeese on crop yields in the Pampas region of Argentina: A contribution for mitigating the conflicts with agriculture. *Agriculture*, *Ecosystems and Environment* 279:33–42. DOI: 10.1016/j.agee.2019.04.002.

Hilje L. 1992. Daño y combate de los roedores plaga en Costa Rica. *Manejo Integrado de Plagas y Agroecología* 23:32–38.

Horrocks JA, Baulu J. 1988. Effects of trapping on the vervet (*Cercopithecus aethiops sabaeus*) population in Barbados. *American Journal of Primatology* 15:223–233. DOI: 10.1002/ajp.1350150305.

Horrocks J, Baulu J. 1994. Food competition between vervets (*Cercopithecus aethiops sabaeus*) and farmers in Barbados: implications for management. *Revue d'Ecologie* 49:281–294.

Horváth A, March IJ, Wolf JHD. 2001. Rodent diversity and land use in Montebello, Chiapas, Mexico. *Studies on Neotropical Fauna and Environment* 36:169–176. DOI: 10.1076/snfe.36.3.169.2130.

Ibañez LM, Andreucci F, Montalti D. 2016. Primer registro de daño a cultivo de frutales por el estornino pinto (*Sturnus vulgaris*) (Passeriformes: Sturnidae) en Argentina. *Acta Zoológica Lilloana*. 60:177–180.

Jackson JE. 1988. Terrestrial mammalian pests in Argentina-an overview. *Proceedings of the Vertebrate Pest Conference* 13:196–198.

Key GE, de la Piedra Constantino R. 1992. The field rat control campaign, Chiapas, Mexico. *Proceedings of the Vertebrate Pest Conference* 15:268–271.

Lima M, Peres CA, Abrahams MI, Junior CAS, Costa GM, Santos RC. 2019. The paradoxical situation of the white-lipped peccary (*Tayassu pecari*) in the state of Mato Grosso, Brazil. *Perspectives in Ecology and Conservation* 17:36–39. DOI: 10.1016/j.pecon.2018.12.001.

Lins PGAS, Ferreira RG. 2019. Competition during sugarcane crop raiding by blond capuchin monkeys (*Sapajus flavius*). *Primates* 60:81–91. DOI: 10.1007/s10329-018-0698-z.

Lobão ESP, Nogueira-Filho SLG. 2011. Human-wildlife conflicts in the Brazilian Atlantic Forest. *Suiform Soundings* 10:14–22.

López-Torres AL, Claudio-Hernández HJ, Rodríguez-Gómez CA, Longo AV, Joglar RL. 2012. Green Iguanas (*Iguana iguana*) in Puerto Rico: Is it time for management? *Biological Invasions* 14:35–45. DOI: 10.1007/s10530-011-0057-0.

Loza-del-Carpio A, Clavitea J, Delgado P. 2016. Incidencia de aves granívoras y su importancia como plagas en el cultivo de quinua (*Chenopodium quinoa* Willd.) en el altiplano Peruano. *Bioagro* 28:139–150.

MacGregor-Fors I, Calderón-Parra R, Meléndez-Herrada A, López-López S, Schondube JE. 2011. Pretty, but dangerous! Records of Monk Parakeets (*Myiopsitta monachus*) in Mexico and their possible invasion effects. *Revista Mexicana de Biodiversidad* 82:1053–1056. DOI: 10.22201/ib.20078706e.2011.3.721.

Marchand G. 2016. Analyse de la dimension spatiale des conflits homme/faune sauvage dans la réserve de développement durable de la rivière Uatumã (Amazonas, Brésil). *Cybergeo: European Journal of Geography* (online). DOI: 10.4000/cybergeo.27807.

McKinney T. 2011. The effects of provisioning and crop-raiding on the diet and foraging activities of human-commensal white-faced Capuchins (*Cebus capucinus*). *American Journal of Primatology* 73:439–448. DOI: 10.1002/ajp.20919.

McKinney T. 2019. Ecological and behavioural flexibility of mantled howlers (*Alouatta palliata*) in response to anthropogenic habitat disturbance. *Folia Primatologica* 90:456–469. DOI: 10.1159/000499825.

Melo C, Cheschini J. 2012. Daños causados por las aves en sorgo (*Sorghum bicolor*) en Brasil central. *Bioagro* 24:33–38.

Mendonça LET, Souto CM, Andrelino, LL, Souto WMS, Vieira WLS, Alves RRN. 2012. Conflitos entre pessoas e animais silvestres no Semiárido paraibano e suas implicações para conservação. *Sitientibus série Ciências Biológicas* 11:185–199. DOI: 10.13102/scb107.

Mitchell B, Bruggers RL. 1985. Aspects of woodpecker damage to cacao in the Dominican Republic. *Tropical Pest Management* 31:148–152. DOI: 10.1080/09670878509370969.

Monge J. 1999. Impacto potencial de la taltuza (*Orthogeomys* sp.) en el cultivo del pejibaye (*Bactris gasipaes*) en Costa Rica. *Agronomía Mesoamericana* 10:133–136. DOI: 10.15517/am.v10i2.17989.

Monge J. 2013. Lista actualizada de aves dañinas en Costa Rica (2012). *UNED Research Journal* 5:111–120. DOI: 10.22458/urj.v5i1.197.

Monge-Meza J, Linares-Orozco J. 2010. Presencia del zorro de cuatro ojos (*Philander opossum*) en el cultivo de piña (*Ananas comusus*). *Agronomía Mesoamericana* 21:343–347. DOI: 10.15517/am.v21i2.4898.

Monge-Meza J. 2011. El impacto de las taltuzas en el cultivo de banano. *Agronomía Mesoamericana* 22:167–174.

Monge-Meza J, Herrera-Murillo F, Arias-Reverón J. 2014. Daños de la rata *Sigmodon hirsutus* (Rodentia: Cricetidae) al cultivo de maní (*Arachis hypogaea*) en Alajuela, Costa Rica. *UNED Research Journal* 6:81–86. DOI: 10.22458/urj.v6i1.318

Naughton-Treves L, Mena JL, Treves A, Alvarez N, Radeloff VC. 2003. Wildlife survival beyond park boundaries: the impact of slash-and-burn agriculture and hunting on mammals in Tambopata, Peru. *Conservation Biology* 17:1106–1117. DOI: 10.1046/j.1523-1739.2003.02045.x

Olivera L, Rodríquez E, Ceretta S, Beyhaut E. 2016. Repelentes de aves aplicados a la semilla de soja: compatibilidad con el inoculante y residualidad en cotiledones. *Agrociencia* 20:51–60.

Parra JG, García AA, Poleo CJ, Fuentes LM. 2012. Aspectos reproductivos y daños causados por una comunidad de roedores en arroz bajo siembra directa en el sistema de riego río Guárico. *Agronomía Tropical* 62:163–170.

Pedrana J, Bernad L, Maceira NO, Isacch JP. 2014. Human-Sheldgeese conflict in agricultural landscapes: Effects of environmental and anthropogenic predictors on Sheldgeese distribution in the southern Pampa, Argentina. *Agriculture*, *Ecosystems and Environment* 183:31–39. DOI: 10.1016/j.agee.2013.09.029.

Pedrosa F, Salerno R, Padilha FVB, Galetti M. 2015. Current distribution of invasive feral pigs in Brazil: Economic impacts and ecological uncertainty. *Natureza e Conservação* 13:84–87. DOI: 10.1016/j.ncon.2015.04.005.

Pereira CZ, Rosa C, Zanzini ACS. 2019. Perception of presence, impact and control of the invasive species *Sus scrofa* in the local community living near the Itatiaia National Park, Brazil. *Ethnobiology and Conservation* 8:1–11. DOI: 10.15451/ec2019-06-8.06-1-11.

Pérez EM, Bulla L. 2000. Dietary relationships among four granivorous doves in Venezuelan savannas. *Journal of Tropical Ecology* 16:865–882. DOI: 10.1017/S0266467400001772.

Pérez E, Pacheco LF. 2006. Damage by large mammals to subsistence crops within a protected area in a montane forest of Bolivia. *Crop Protection* 25:933–939. DOI: 10.1016/j.cropro.2005.12.005.

Pérez E, Pacheco LF. 2014. Mitigación de daños provocados por fauna silvestre en cultivos agrícolas en un bosque montano de Bolivia. *Revista de Biología Tropical* 62:1495–1507.

Peyton B. 1980. Ecology, distribution, and food habits of spectacled bears, *Tremarctos ornatus*, in Peru. *Journal of Mammalogy* 61:639–652. DOI: 10.2307/1380309.

Poleo CJ, Fuentes L, Vivas L. 2010. Caracterización reproductiva de una población de *Zygodontomys brevicauda* (Rodentia: Cricetidae) capturada en siembras de arroz del estado Guárico, Venezuela. *Agronomía Tropical* 60:43–48.

Ranvaud R, de Freitas KC, Bucher EH, Dias HS, Avanzo VC, Alberts CC. 2001. Diet of Eared Doves (*Zenaida auriculata*, Aves, Columbidae) in a sugar-cane colony in South-eastern Brazil. *Brazilian Journal of Biology* 61:651–660. DOI: 10.1590/S1519-69842001000400015

Renfrew RB, Saavedra AM. 2007. Ecology and conservation of Bobolinks (*Dolichonyx oryzivorus*) in rice production regions of Bolivia. *Ornitología Neotropical* 18:61–74*.*

Renfrew RB, Hill JM, Kim DH, Romanek C, Perlut NG. 2017. Winter diet of Bobolink, a long-distance migratory grassland bird, inferred from feather isotopes. *The Condor* 119:439–448. DOI: 10.1650/CONDOR-16-162.1.

Robles J, Jacobsen SE, Rasmussen C, Otazu V, Mandujano J. 2003. Plagas de aves en Quinua (*Chenopodium quinoa* Willd.) y medidas de control en el Perú central. *Revista Peruana de Entomología*. 43:147–151.

Rocha LC, Fortes VB. 2015. Perceptions and attitudes of rural residents towards capuchin monkeys, in the area of influence of the Dona Francisca hydroelectric power plant, South Brazil. *Ambiente e Sociedade* 18:19–34. DOI: 10.1590/1809-4422ASOC825V1842015

Rodriguez EN, Avery ML. 1996. *Agelaius* blackbirds and rice in Uruguay and the southeastern United States. *Proceedings of the Seventeenth Vertebrate Pest Conference* 17:94–98.

Rodriguez EN, Bruggers RL, Bullard RW, Cook R. 1995. An integrated strategy to decrease eared dove damage in sunflower crops. *National Wildlife Research Center Repellents Conference* 409–421.

Rodriguez EN, Tiscornia G, Tobin ME. 2004. Bird depredations in Uruguayan vineyards. *Proceedings of the Vertebrate Pest Conference* 21:136–139.

Romero-Balderas KG, Naranjo EJ, Morales HH, Nigh RB. 2006. Daños ocasionados por vertebrados silvestres al cultivo de maíz en la Selva Lacandona, Chiapas, México. *Interciencia* 31:276–283.

Rosa CA, Wallau MO, Pedrosa F. 2018. Hunting as the main technique used to control wild pigs in Brazil. *Wildlife Society Bulletin* 42:111–118. DOI: 10.1002/wsb.851.

Sánchez R, Ballari SA, Bucher EH, Masello JF. 2016. Foraging by burrowing parrots has little impact on agricultural crops in northeastern Patagonia, Argentina. *International Journal of Pest Management* 62:326–335. DOI: 10.1080/09670874.2016.1198061.

Sánchez-Cordero V, Martínez-Meyer E. 2000. Museum specimen data predict crop damage by tropical rodents. *Proceedings of the National Academy of Sciences* 97:7074–7077. DOI: 10.1073/pnas.97.13.7074

Santos GC. 2018. Characteristics of rodent outbreaks in the low San Francisco Sergipano (Sergipe, Brazil) and influence of anomalies on sea surface temperature on temperatures in this region. *International Journal of Design and Nature and Ecodynamics* 13:156–165. DOI: 10.2495/DNE-V13-N2-156-165.

Saucedo Castillo OM, Fernández Pérez LE, Quiñones Ramos R, Rodríguez Valdés G, Moya Álvarez A. 2017. Las aves granívoras y el cultivo del sorgo en la provincia de Villa Clara, Cuba. *Revista Centro Agrícola* 44:36–43.

Silva-Andrade HL, de Andrade LP, Muniz LS, Telino-Júnior WR, Albuquerque UP, Lyra-Neves RM. 2016. Do farmers using conventional and non-conventional systems of agriculture have different perceptions of the diversity of wild birds? Implications for conservation. *PLoS ONE* 11:e0156307. DOI: 10.1371/journal.pone.0156307.

Silva-Rodríguez EA, Ortega-Solís GR, Jiménez JE. 2006. Aves silvestres: actitudes, prácticas y mitos en una localidad rural del sur de Chile. *Boletín Chileno de Ornitología* 12:2–14.

Spagnoletti N, Cardoso TCM, Fragaszy D, Izar P. 2017. Coexistence between humans and capuchins (*Sapajus libidinosus*): comparing observational data with farmers’ perceptions of crop losses. *International Journal of Primatology* 38:243–262. DOI: 10.1007/s10764-016-9926-9.

Trivedi MR, Cornejo FH, Watkinson AR. 2004. Seed predation on brazil nuts (*Bertholletia excelsa*) by macaws (Psittacidae) in Madre de Dios, Peru. *Biotropica* 36:118–122. DOI: 10.1646/03050.

Valencia D. 1980. Rat control in coconut palms in Colombia. *Proceedings of the Vertebrate Pest Conference* 9:110–113.

Valencia D, Elias DJ, Ospina JA. 1994. Rodent pests in Colombian agriculture*. Proceedings of the Vertebrate Pest Conference* 16: 92–94.

Villa CB, Lopez-Forment W, Villa CM, Prescott CV. 1998. Not all sigmodontine rodents in the sugarcane fields in coastal Veracruz, Mexico, are pests. *Proceedings of the Vertebrate Pest Conference* 18:236–241. DOI: 10.5070/V418110096.

Villafaña FM, Pupo MS, Blanco JR, Sánchez Rojas LG, Campos Muñoz C. 1999. Evaluación del impacto del biorrodenticida Biorat en poblaciones de roedores establecidos en varios cultivos en la República de Costa Rica. *Revista Cubana de Medicina Tropical* 51:185–188.

del Villar-González D. 2000. Principales vertebrados plaga en México: situación actual y alternativas para su manejo. *Revista Chapingo Serie Ciencias Forestales y del Ambiente* 6:41–54.

Waters S. 2015. Crop-raiding Baird’s tapir provoke diverse reactions from subsistence farmers in Belize. *Tapir Conservation* 24:8–10. DOI: 10.5281/zenodo.22642.
